# Noncommutative Biology: Sequential Regulation of Complex Networks

**DOI:** 10.1371/journal.pcbi.1005089

**Published:** 2016-08-25

**Authors:** William Letsou, Long Cai

**Affiliations:** Division of Chemistry and Chemical Engineering, California Institute of Technology, Pasadena, California, United States of America; Stony Brook University, UNITED STATES

## Abstract

Single-cell variability in gene expression is important for generating distinct cell types, but it is unclear how cells use the same set of regulatory molecules to specifically control similarly regulated genes. While combinatorial binding of transcription factors at promoters has been proposed as a solution for cell-type specific gene expression, we found that such models resulted in substantial information bottlenecks. We sought to understand the consequences of adopting sequential logic wherein the time-ordering of factors informs the final outcome. We showed that with noncommutative control, it is possible to independently control targets that would otherwise be activated simultaneously using combinatorial logic. Consequently, sequential logic overcomes the information bottleneck inherent in complex networks. We derived scaling laws for two noncommutative models of regulation, motivated by phosphorylation/neural networks and chromosome folding, respectively, and showed that they scale super-exponentially in the number of regulators. We also showed that specificity in control is robust to the loss of a regulator. Lastly, we connected these theoretical results to real biological networks that demonstrate specificity in the context of promiscuity. These results show that achieving a desired outcome often necessitates roundabout steps.

## Author Summary

DNA is the blueprint of life. Yet the order in which a cell follows these instructions makes it capable of generating thousands of different fates. How this information is extracted from underlying gene regulatory networks is unclear, especially given that biological networks are highly interconnected, and that the number of signaling pathways is relatively small (approximately 5–10). The conventional approach for increasing the information capacity of a limited set of regulators is to use them in combination. Surprisingly, combinatorial logic does not increase the diversity of target configurations or cell fates, but instead causes information bottlenecks. A different approach, called sequential logic, uses noncommutative sequences of a small set of regulators to drive networks to a large number of novel configurations. If certain targets are first protected, then even promiscuous regulators can activate specific subsets of lineage-specific targets. In this paper we show how sequential logic outperforms combinatorial logic, and argue that noncommutative sequences underlie a number of cases of biological regulation, e.g. how a small number of signaling pathways generates a large diversity of cell types in development. In addition to explaining biological networks, sequential logic may be a general experimental design strategy in synthetic and single-cell biology.

## Introduction

A fundamental question in systems biology is how a small number of signaling inputs specifies a large number of cell fates through the coordinated expression of thousands of genes. This problem is especially challenging given that gene regulatory and other types of networks in biology tend to be highly interconnected and their regulators promiscuous, with regulators affecting multiple targets and targets being affected by multiple regulators. Examples of this architecture include: transcription factor binding networks in bacteria [1], yeast [2, 3], plants [4], and animals [5, 6]; cellular signalling pathways involved in growth and differentiation [7–9]; the interactome of protein kinases and phosphatases [10, 11]; and synaptic connections between different layers of the brain [12]. Furthermore, because the targets and regulators are often well-mixed and mutually accessible in the cell, most actions are likely to have nonspecific and undesired effects.

At the same time, regulatory molecules drive networks to a large number of highly specific outcomes or cell fates. Although there are approximately four hundred canonical cell types in the adult human [13], recent single-cell RNA expression profiling experiments in the developing embryo [14, 15], brain [16], hematopoietic system [17, 18], and other organs [19, 20], have indicated that there may be thousands more.

**Fig 1:**
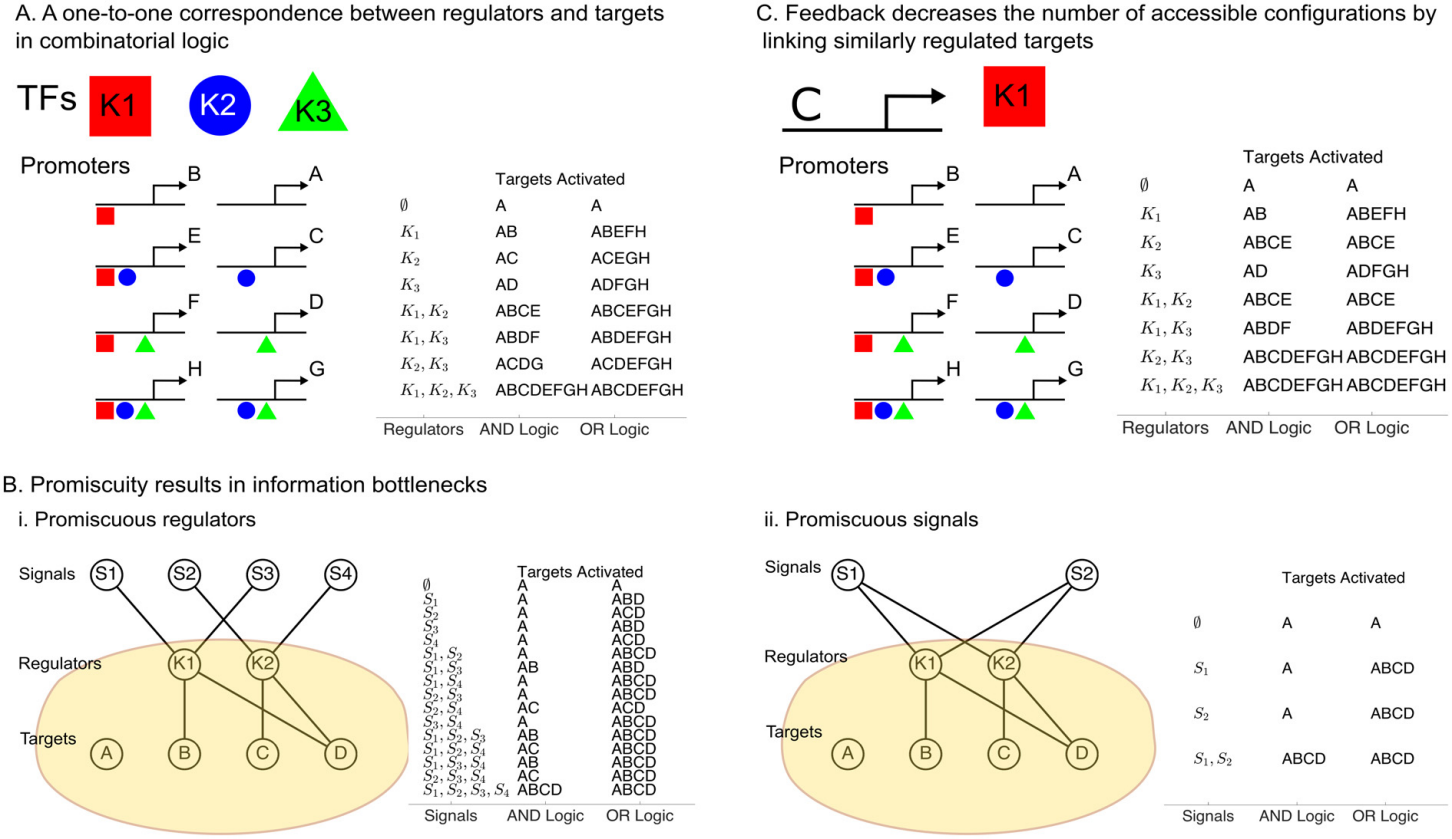
Combinatorial logic bottlenecks information flow in networks. **(A)** The number of ways that three TFs (*K*_1_, *K*_2_, *K*_3_) can be ON or OFF (tabulated at right) is the same as the number of ways they can bind at promoters (left). An equal number of gene expression states are observed whether the TFs use AND logic (requiring all factors be present) or OR logic (requiring at least one of the factors). **(B)** Signal-to-target information flow is bottlenecked by regulators if (i) the regulators respond to multiple targets, or (ii) the signals activate multiple regulators. The allowed target states are tabulated for signals using AND logic and regulators using AND/OR logic. **(C)** A feedback loop causes constitutive activation of a regulator (*K*_1_) and leads to fewer accessible configurations (tabulated at right).

Given there are only a few signaling pathways used in metazoan development [21, 22], understanding how cells reach their final outcomes when there are fewer regulators than fates and/or targets is an unsolved problem. One extensively studied solution for the control of promiscuous gene networks is combinatorial binding of DNA-binding transcription factors (TFs) at the promoter [23–31]. At the level of individual promoters, combinatorial binding ensures that individual genes are ON only when specific combinations of TFs are present (Fig 1A). However, on the genome level, combinatorial regulation restricts which sets of genes may be ON at the same time. For example, using AND logic, gene H in Fig 1A is only ON in the case that the three TFs *K*_1_, *K*_2_, and *K*_3_ are concurrent at the H promoter; but these stringent requirements mean that H can never be transcribed independently of the less highly-regulated genes A-G. (A similar conclusion holds for OR logic.)

In fact, using combinatorial control, there is a one-to-one correspondence between configurations of the targets and configurations of the regulators. As shown in Fig 1A, the ON/OFF states of 3 TFs uniquely define the binding combinations at 2^3^ = 8 promoters. A similar conclusion holds when the regulators are expressed in a graded fashion.

This one-to-one correspondence is the fundamental limitation of combinatorial regulation: it requires an equal number of regulators and independently controlled targets and/or cell fates. Applied to embryonic development, combinatorial control requires that hundreds or thousands of cell-type specific TF combinations be generated in a spatially precise manner at the start. However, the combinatorial scheme does not explain how the TF states are regulated in the first place, and thus it offers no new insight into how cell fate is specified.

The limitations of combinatorial logic can also be understood from an information theoretic point of view. In particular, it is impossible to specify arbitrary cell fates if the regulatory layer bottlenecks the capacity of the targets to receive messages from extracellular signals. It is known that some ten to twenty types of signals [21, 22] converge onto membrane-bound regulators in many different combinations, permitting messages to be passed to the downstream targets. Much of this information stands to be lost, however, if the network relies on combinatorial logic alone: the regulatory layer simply cannot transmit messages in their entirety if there are more signals than regulators. Thus, combinatorial logic strongly circumscribes what fates are ultimately reachable. Cell fate information is lost not only if the signals are more numerous than the regulators, but also if the connections between signals and regulators are promiscuous (Fig 1B). When different signals activate the same regulators (Fig 1Bi), certain signaling inputs become redundant. On the other hand, when same signal activates different regulators (Fig 1Bii), some of the regulators become redundant. One may determine by direct enumeration exactly how redundancy decreases the number of configurations available to the targets (Materials and Methods Sections 1 and 2). These preliminary conclusions are at odds with the observation that signaling molecules are deployed over time in a complex code [32]. How do these messages in the signal space reach the targets if the regulatory layer imposes a bottleneck on information flow?

In addition, feedback regulation—a common feature of regulatory networks—exacerbates information bottlenecks when coupled with combinatorial logic. Stated another way, feedback merely widens the basin of attraction of certain promoter configurations at the expense of the number of distinct configurations. In Fig 1C, constitutive expression of *K*_1_ by C means that C is never ON independently of the targets regulated by *K*_1_. Thus, the number of accessible configurations decreases from 8 to 6 without allowing new target configurations to be explored.

We need an alternative to combinatorial logic in cell fate specification that overcomes information bottlenecks. Here, we considered time-ordered control schemes, which we refer to as sequential logic. In this scheme, regulators can be applied in a stepwise manner; the entire sequence matters, so the final configurations can differ if the same regulators are permuted in time. In order for different temporal sequences to carry distinct information, the actions of the regulators must be *noncommutative*. This is the case, for example, when a regulator protects its targets from the action of another regulator, as when loci recruited to repressive chromatin compartments are protected from further modification [33, 34].

While it is not surprising that noncommutative sequences like this result in different outcomes at the single promoter level, these simple mechanisms may have nontrivial implications for regulation at the genome level. In particular, noncommutativity permits the same regulators to be used at different times with distinct effects. This is seen in development when ubiquitous signaling molecules like FGF family members exert different effects depending on the time and context of their expression [35–38]. Reuse of factors could greatly expand the information capacity of the major signaling pathways.

A number of examples show that noncommutativity may be a general strategy in other areas of biology. In hematopoietic stem cells, activation of GATA2 and C/EBP*α* in different orders results in different cell fates [39]. In neurobiology, different temporal orderings of the same inputs lead to distinct firing patterns [40–42]. In the field of synthetic biology, a DNA switch was developed that could detect the order in which invertase enzymes were applied [43]. And in evolutionary biology, the order in which mutations arise was recently implicated in determining a genotype’s fitness [44–47]. There is also accumulating evidence for sequential logic in transcriptional control: signaling molecules and TFs in mammalian cells, including ERK [48], NF-*κ*B [49, 50], p53 [51], as well as in yeast [52–54] have been observed to pulse, suggesting that TF timing may be used to control the transcriptional state of the cell.

By applying sequential logic, we show that, even in complex and promiscuously regulated networks, specific target configurations can be reached using a temporal sequence of regulators. In particular, we consider two models inspired by (i) kinase/neural networks and (ii) chromosome folding and show analytically that both scale super-exponentially. We further show that noncommutative networks are robust to the loss of regulators, suggesting a mechanism for regulator evolution. We also show that regulators induce different orbits in expression space, which is related to the number of networks that can be controlled in parallel. We conclude by discussing how these models apply to interconnected networks in and outside biology and by providing possible experimental tests of the theoretical concepts. Theorems and proofs are given in the Materials and Methods.

## Results

### A time-sequence ratchet model generates more diversity than combinatorial logic in multiply-connected networks

**Fig 2:**
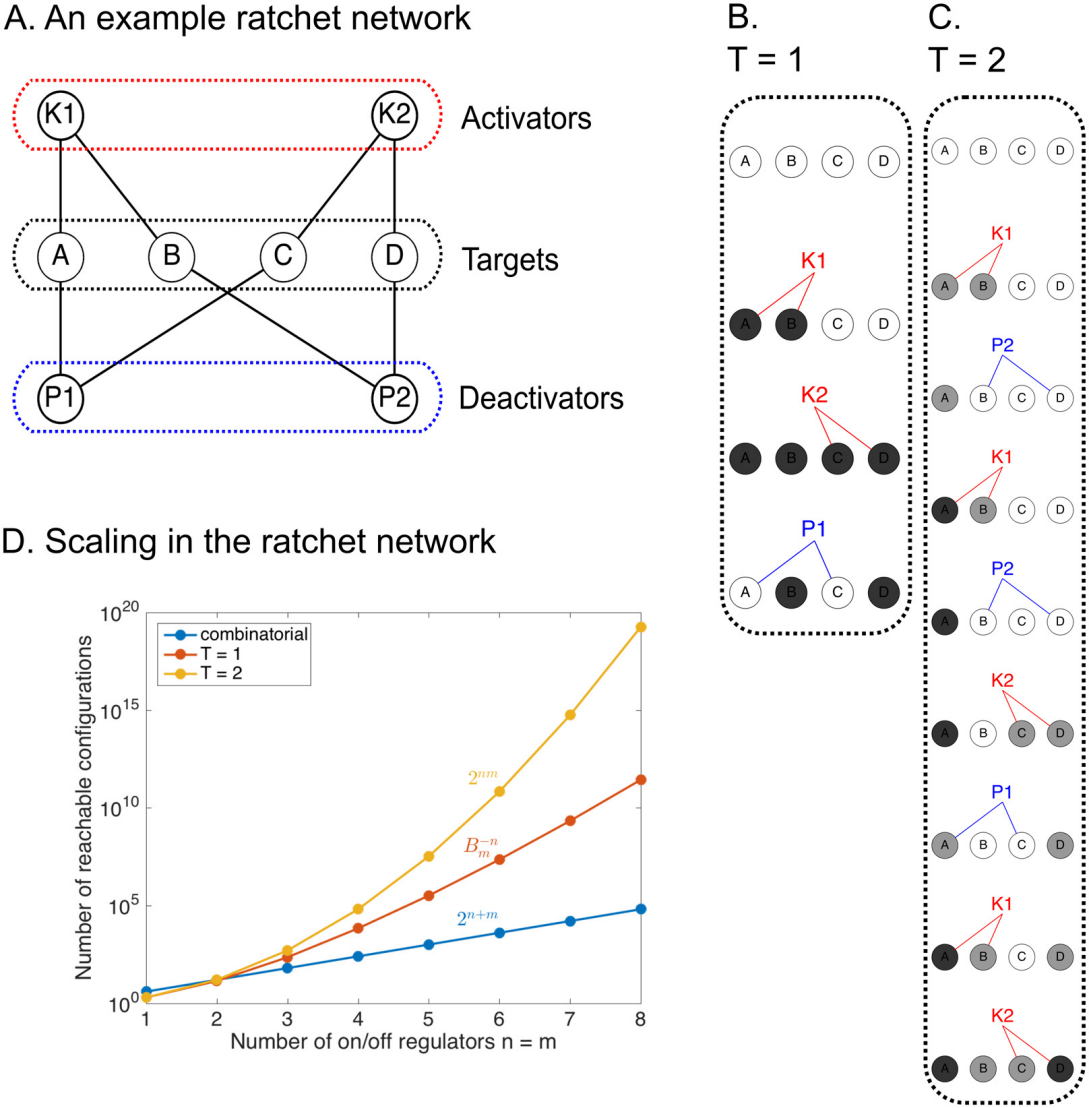
The ratchet model attains configurations not reachable by combinatorial logic. **(A)** The ratchet model for *n* = *m* = 2 and *l*_*n*_ = *l*_*m*_ = 1. Activators (*K*’s) and deactivators (*P*’s) turn targets ON and OFF, respectively. **(B)** An example temporal sequence for the network with a threshold equal to 1. Black targets are in the 1 state. **(C)** An example sequence for the same network with threshold equal to 2. Gray targets are in the 1 state, and black targets are in the 2 state. **(D)** Scaling laws for the threshold *T* = 1 (red) and *T* = 2 (yellow) are shown for symmetric networks (*n* = *m*). A comparison to combinatorial logic with an equivalent number of regulators (*n* + *m*) is shown in blue.

To consider how time-ordered sequences of regulators can specifically control groups of targets, we begin by analyzing a generic two-layer network that is an extension of combinatorial logic (Fig 2, Materials and Methods Sections 1 and 2). In this model, each regulator controls multiple targets, and each target is accessible to any of its regulators. The model is meant to be analogous to the cellular environment wherein regulators and targets are well-mixed. For example, targets could be substrate proteins capable of multi-site phosphorylation [55, 56], and regulators the kinases and phosphatases. Targets could also be neurons and regulators their upstream excitatory and inhibitory inputs [12]. We denote by *K* the set of activators (i.e. kinases) and *P* the set of deactivators (i.e. phosphatases). Each target has a ladder of (integer-valued) *states*, and together the states of the targets are a *configuration* of the network. (This distinction is in contrast to the common usage of “state” as a gene expression vector.) An additional parameter, the threshold *T*, determines the number of rungs on the ladder. Regulators ratchet the targets through their states, and only targets that have reached threshold will be ON at the end of a sequence of regulators. If each target in the group can be controlled by a unique combination of *K*’s and *P*’s, what ON/OFF configurations are possible?

In this model, termed the ratchet network (Fig 2A), each of *n* *K*’s and *m* *P*’s control N=(nln)(mlm) unique targets, with the connectivity parameters *l*_*n*_ and *l*_*m*_ specifying the number of regulators to which each target connects. Consider the sequence *K*_1_ *K*_2_ *P*_1_ acting on the targets *A*, *B*, *C*, and *D* (Fig 2B). In the final configuration, *B* and *D* are ON together even though no single *K* connects to both, and *A* and *C* are OFF, even though both share and activator with *B* and *D*. Therefore, this simple model illustrates the important point that similarly regulated targets can be in independently controlled using sequential logic.

With threshold *T* = 1, not all configurations are reachable. Observe that there is no way to specifically activate *A* and *D* while leaving *B* and *C* OFF. This result is surprising given that *A* and *D* share no regulators: specificity depends on the network as a whole, not just individual targets. By going to *T* = 2, the forbidden configuration becomes accessible (Fig 2C), along with all ON/OFF states (below).

### A combinatorial formulation of the model as a connectivity matrix

The model described above can be formalized as a combinatorial object that we refer to as the *connectivity matrix* **A**. This formulation is useful because it is amenable to studying scaling, and it permits a direct comparison between noncommutative ratchet networks and standard combinatorial logic. For the interested reader, the models considered in this paper have a universal formulation as noncommutative matrix operators on the vector space of configurations (Materials and Methods Section 9).

Typically, the state of *N* targets is represented as an *N*-dimensional vector. If each target is controlled by a unique (*K*_*i*_, *P*_*j*_) pair (i.e. *l*_*n*_ = *l*_*m*_ = 1), the *N* = *nm*-dimensional vector can be re-formulated as an *n* × *m* matrix

$$A=\begin{array}{cc} & \begin{array}{ccc} P_{1} & \cdots & P_{m} \end{array}\\ \begin{array}{c} K_{1}\\ \vdots \\ K_{n} \end{array} & \left(\begin{array}{ccc} A_{1,1} & \cdots & A_{1,m}\\ \vdots & \ddots & \vdots \\ A_{n,1} & \cdots & A_{n,m} \end{array}\right) \end{array}$$ (1)

where each entry **A**_*i*,*j*_ ∈ {0, 1, …, *T*} is the state of the target regulated by *K*_*i*_ and *P*_*j*_. For example, the connectivity matrix for the network in Fig 2 is

$$A=\begin{array}{cc} & \begin{array}{cc} P_{1} & P_{2} \end{array}\\ \begin{array}{c} K_{1}\\ K_{2} \end{array} & \left(\begin{array}{cc} A & B\\ C & D \end{array}\right) \end{array}.$$ (2)

In general, a regulator may connect to multiple targets (i.e. *l*_*n*_, *l*_*m*_ > 1, see below), so that each entry of **A** may be thought of as an *M*-dimensional vector (*M* determined in Materials and Methods Section 1). It turns out that this is an unnecessary complication; we instead let each **A**_*i*,*j*_ = 1 if at least one of the *M* targets regulated by *K*_*i*_ and *P*_*j*_ is ON, and **A**_*i*, *j*_ = 0 only if all *M* targets are OFF.

In this formulation *K*_*i*_ and *P*_*j*_ are raising and lowering operators that map *n* × *m* matrices to *n* × *m* matrices via the rules

$$\begin{array}{cc} K_{i}\left(A_{i,j}\right) & =\left\{\begin{array}{cc} A_{i,j}+1 & \;\text{if}\;A_{i,j}<T\\ A_{i,j} & \;\text{if}\;A_{i,j}=T \end{array}\right.\\ P_{i}\left(A_{i,j}\right) & =\left\{\begin{array}{cc} A_{i,j}-1 & \;\text{if}\;A_{i,j}>0\\ 0 & \;\text{if}\;A_{i,j}=0. \end{array}\right. \end{array}$$ (3)

From Eq (3), any sequence *K*_*i*_1__ *K*_*i*_2__⋯*K*_*i*_*k*__ of all *K*’s is commutative, because any target controlled by *t* ≤ *k* of the *K*’s will be in state *t* ≤ *T* at the end of the sequence, regardless of the order. A similar argument holds for the *P*’s. However, sequences consisting of both *K*’s and *P*’s are in general noncommutative. This is due to edge effects when **A**_*i*,*j*_ = 0 or *T*. If **A**_*i*,*j*_ = *T*, for example, then *K*_*i*_ *P*_*j*_ results in **A**_*i*,*j*_ = *T* − 1, whereas *P*_*j*_ *K*_*i*_ gives **A**_*i*,*j*_ = *T*. Therefore, **A** gives insight into both the configuration of the targets and the noncommutativity of the regulators.

The problem of determining the number of accessible configurations in a network is reduced to finding the number of matrices satisfying certain patterns. For example, combinatorial logic with *T* = 1 corresponds to the special case in which the only sequences are the 2^*n*^ combinations of the *n* *K*’s. In an *n* × 1 connectivity matrix, activating *K*_*i*_ corresponds to turning all 0’s in row *i* into 1’s. There are 2^*n*^ matrices generated by this procedure. More complicated cases of combinatorial logic can be studied this way (Materials and Methods Section 2), but it turns out that the total number of network configurations is always less than 2^*n* + *m*^, with *n* + *m* the total number of regulators. This is important because noncommutative models can bypass the exponential limit.

### The ratchet model scales as the poly-Bernoulli numbers

We used the connectivity matrix representation of the ratchet network to determine the scaling as function of the number of regulators *n* and *m*, with each target connected to a unique (*K*, *P*) pair (i.e. *l*_*n*_ = *l*_*m*_ = 1) and the threshold *T* = 1. *K*_*i*_ turns 0’s to 1’s in row *i* and *P*_*j*_ turns 1’s to 0’s in column *j*. The rules are consistent with the one-pot reaction model in which all substrates receptive to *K*_*i*_ are promoted when *K*_*i*_ is active. For example, the sequence *K*_1_ *K*_2_ *P*_1_ in Fig 2B can be recast as

$$\begin{array}{c} \left(\begin{array}{cc} 0 & 0\\ 0 & 0 \end{array}\right)\overset{K_{1}}{\to }\left(\begin{array}{cc} 1 & 1\\ 0 & 0 \end{array}\right)\overset{K_{2}}{\to }\left(\begin{array}{cc} 1 & 1\\ 1 & 1 \end{array}\right)\overset{P_{1}}{\to }\left(\begin{array}{cc} 0 & 1\\ 0 & 1 \end{array}\right). \end{array}$$ (4)

The main result is that **A** must avoid the patterns $\left(\begin{array}{cc} 1 & 0\\ 0 & 1 \end{array}\right)$ and $\left(\begin{array}{cc} 0 & 1\\ 1 & 0 \end{array}\right)$ in any 2 × 2 sub-block (Materials and Methods Section 3). Brewbaker [57] enumerated the *n* × *m* binary matrices avoiding these patterns and showed that they scale as the poly-Bernoulli numbers [58]

$$\begin{array}{c} B_{m}^{-n}=B_{n}^{-m}=\sum_{j=0}^{m}{\left(-1\right)}^{\left(n+j\right)}j!{\left(j+1\right)}^{n}\left\{\begin{array}{c} n\\ j \end{array}\right\}=\sum_{j=0}^{\text{min}\left(n,m\right)}{\left(j!\right)}^{2}\left\{\begin{array}{c} m+1\\ j+1 \end{array}\right\}\left\{\begin{array}{c} n+1\\ j+1 \end{array}\right\}, \end{array}$$ (5)

where $\left\{\begin{array}{l} n\\ j \end{array}\right\}$ is a Stirling number of the second kind, defined combinatorially as the number of ways to put *j* labelled balls into *n* unlabelled boxes such that no box is empty [59]. These numbers scale not quite as fast as 2^*N*^ = 2^*nm*^, but much faster than 2^*n* + *m*^, the maximum number of states in the equivalent combinatorial network (Fig 2D). Thus, a simple time-sequence model is able to generate super-exponential scaling.

### All binary ON/OFF states are reachable for an increased threshold

Are more configurations accessible if multiple activation events are needed before reaching threshold? For example, neurons require the summation of multiple excitatory inputs to reach action potential, and proteins need to be phosphorylated at multiple sites before they are activated [55, 56]. We found that by increasing the threshold to *T* = 2, all 2^*N*^ ON/OFF configurations of the *N* targets become reachable. In the connectivity matrix formulation, $\left(\begin{array}{cc} 1 & 0\\ 0 & 1 \end{array}\right)$ and $\left(\begin{array}{cc} 0 & 1\\ 1 & 0 \end{array}\right)$ are no longer forbidden, which we show with an inductive proof (Materials and Methods Section 4). This scaling law (Fig 2D), achieves the maximum of reachability and specificity; it far exceeds the scaling 2^*n* + *m*^ of the combinatorial model.

Being able to reach the entire ON/OFF space of *N* targets is overkill for most biological networks, which only display a relatively small number of stable configurations. The major implication of this result is that multiple levels of activity permit more targets to be controlled independently.

### Increased regulatory connectivity generates robustness

As sequential logic allows a large number of configurations to be reached in a complex network, we asked whether increasing the connectivity of the network (*l*_*n*_ and *l*_*m*_) can maintain the specificity of the network while making it robust to the loss of a regulator. This is potentially relevant to evolution of biological networks, because redundant connections allow the network to repurpose regulators for new functions without severely impairing existing ones [60].

**Fig 3:**
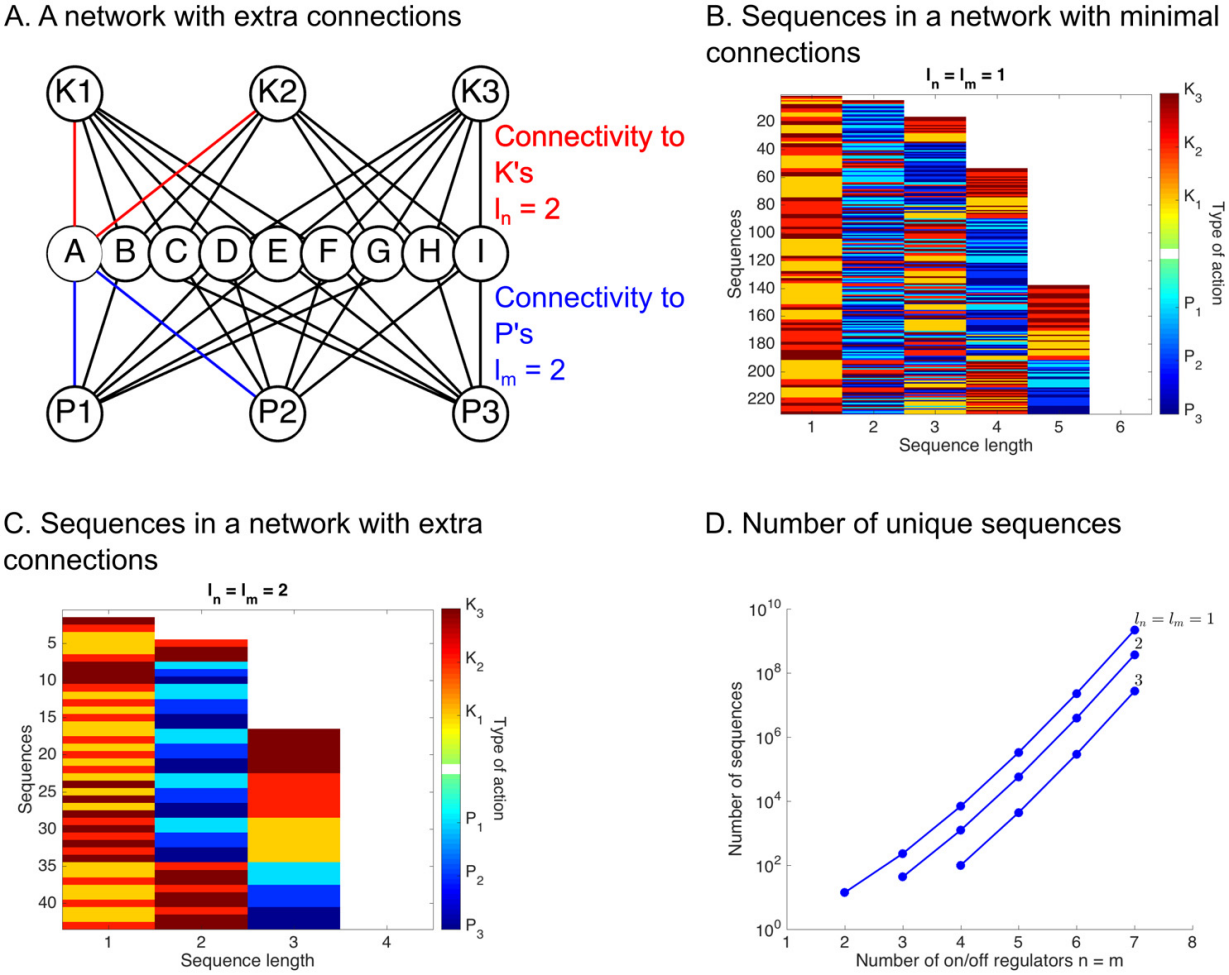
Multiple connections in the ratchet network decreases the number of configurations. **(A)** An example network where each target has *l*_*n*_ = 2 connections to the *K*’s (red) and *l*_*m*_ = 2 connections to the *P*’s (blue). **(B)** A list of the minimal length sequences generating unique configurations in the network in when *l*_*n*_ = *l*_*m*_ = 1. Red bars are *K* actions and blue bars are *P* actions. **(C)** The list of minimal length sequences when *l*_*n*_ = *l*_*m*_ = 2. Some sequences now map to the same configuration. **(D)** Analytical solution for the number of sequences as a function of *n* = *m* for different *l*_*n*_ = *l*_*m*_ families.

In the ratchet model, an increase in the connectivity parameters to *l*_*n*_ = 2 *K*’s and *l*_*m*_ = 2 *P*’s permits multiple targets to share a common (*K*, *P*) pair (Fig 3A). The connectivity matrix incorporating the extra links in the network in Fig 3A is

$$\begin{array}{l} \begin{array}{cccc} & \;P_{1} & \;P_{2} & \;P_{3} \end{array}\\ A=\begin{array}{c} K_{1}\\ K_{2}\\ K_{3} \end{array}\left(\begin{array}{ccc} ABDE & ACDF & CDEF\\ ABGH & ACGI & BCHI\\ DEFG & DFGI & EFHI \end{array}\right). \end{array}$$ (6)

Now that each entry of **A** is a group of *M* > 1 targets, it makes sense to track the state of the group as a whole with a single number **A**_*i*,*j*_. Even though a target appears in multiple entries of **A**, the rules prevent a regulator from altering the state of groups at remote locations (e.g. *K*_1_ cannot change the state of the group at **A**_2, 2_).

We prove in the Materials and Methods that all sequences using at least *n* − *l*_*n*_ + 1 *K*’s and *m* − *l*_*m*_ *P*’s are redundant with shorter sequences (Fig 3B and 3C, Materials and Methods Section 5). For example, the sequences *K*_1_ *K*_2_ *K*_3_ is required to turn ON all targets in the case *l*_*n*_ = *l*_*m*_ = 1, but if *l*_*n*_ = *l*_*m*_ = 2, the shorter sequences *K*_1_ *K*_2_, *K*_1_ *K*_3_, and *K*_2_ *K*_3_ have the same effect. We derived a recursive formula that eliminates the redundant sequences in each (*n*, *m*, *l*_*n*_, *l*_*m*_) instance to derive the number of sequences in (*n*, *m*, *l*_*n*_ + 1, *l*_*m*_) and (*n*, *m*, *l*_*n*_, *l*_*m*_ + 1) (Fig 3D and S2 Fig). The formula agreed exactly with an algorithm designed to find all minimal length sequences (Materials and Methods 5). Notably, increasing *l*_*n*_, *l*_*m*_ reduced the number of configurations. We observed a similar effect in combinatorial logic (S1 Fig).

**Fig 4:**
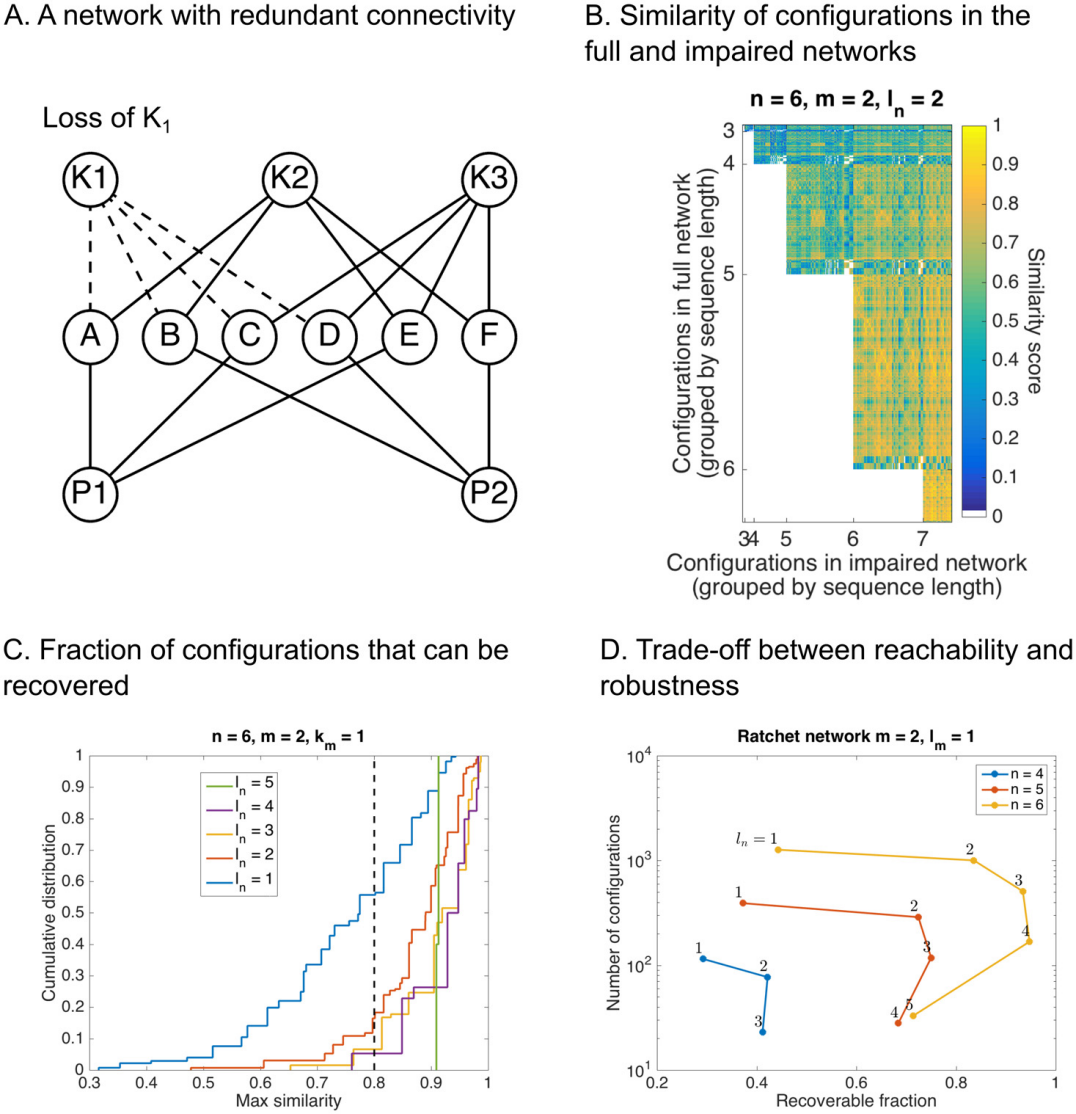
The ratchet network is robust to loss of a regulator. **(A)** A schematic illustration of the experiment. The regulator *K*_1_ was deleted from networks with *m* = 2 *P*’s and variable *n* for different values of the connectivity *l*_*n*_. The resulting number of configurations was computed by simulation. **(B)** Correlation coefficient between configurations in the full network (all *K*’s; rows) and the impaired network (without *K*_1_; columns). All rows with exact matches were deleted. **(C)** Cumulative distribution *F*(*x*) of the maximum correlation coefficient *x* for each row in **C** for different values of *l*_*n*_. The dashed line is the similarity cutoff 0.8. **(D)** Tradeoff between reachability and robustness. The number of reachable configurations as a function of (*n*, *l*_*n*_) is plotted vs. the fraction of states above the similarity cutoff 0.8 (i.e. 1 − *F*(0.8)) for different values of *n*.

To investigate the robustness of sequential logic networks, we studied the effect of deleting regulators in increasingly connected networks on the number of reachable configurations (Fig 4A). We hypothesized that sequences that activate similar subsets of targets should be able to recoup permanently lost configurations. To test this, we computed the normalized correlation coefficient between configurations in the network using all *K*’s (the full network) and configurations in the network without *K*_1_ (the impaired network), subject to the constraint that those configurations were reached using longer sequences (Fig 4B). To focus on the recoverable fraction, we deleted all configurations that had an exact match. Highly similar configurations (yellow) clustered to the right of the plot, indicating that longer sequences can be used to recover lost configurations.

How similar are the recouped configurations? As connectivity increased, the maximum similarity became increasingly concentrated above 0.8 (Fig 4C). There is generally a tradeoff between reachability and the size of the fraction above 0.8 (Fig 4D). The tradeoff is nonlinear, however: using *l*_*n*_ = 2 gave the greatest increase recoverability for the smallest loss of configurations, showing that an intermediate level of redundancy can buffer the network to loss of regulators. The above analyses demonstrate that specificity of control is not compromised when regulators are lost or repurposed in heavily interconnected networks.

### Sequestration networks generate diversity through protected states

In the ratchet model, all targets are accessible to their regulators at all times. However, in some cases targets may be shielded from regulators: for example, genes can be silenced by sequestration in various nuclear compartments [61, 62]. This was seen in a landmark study by Filion *et al* [63], who used a DNAse accessibility assay to show that genes associate with different regulators depending on their chromatin “color” or accessibility status.

**Fig 5:**
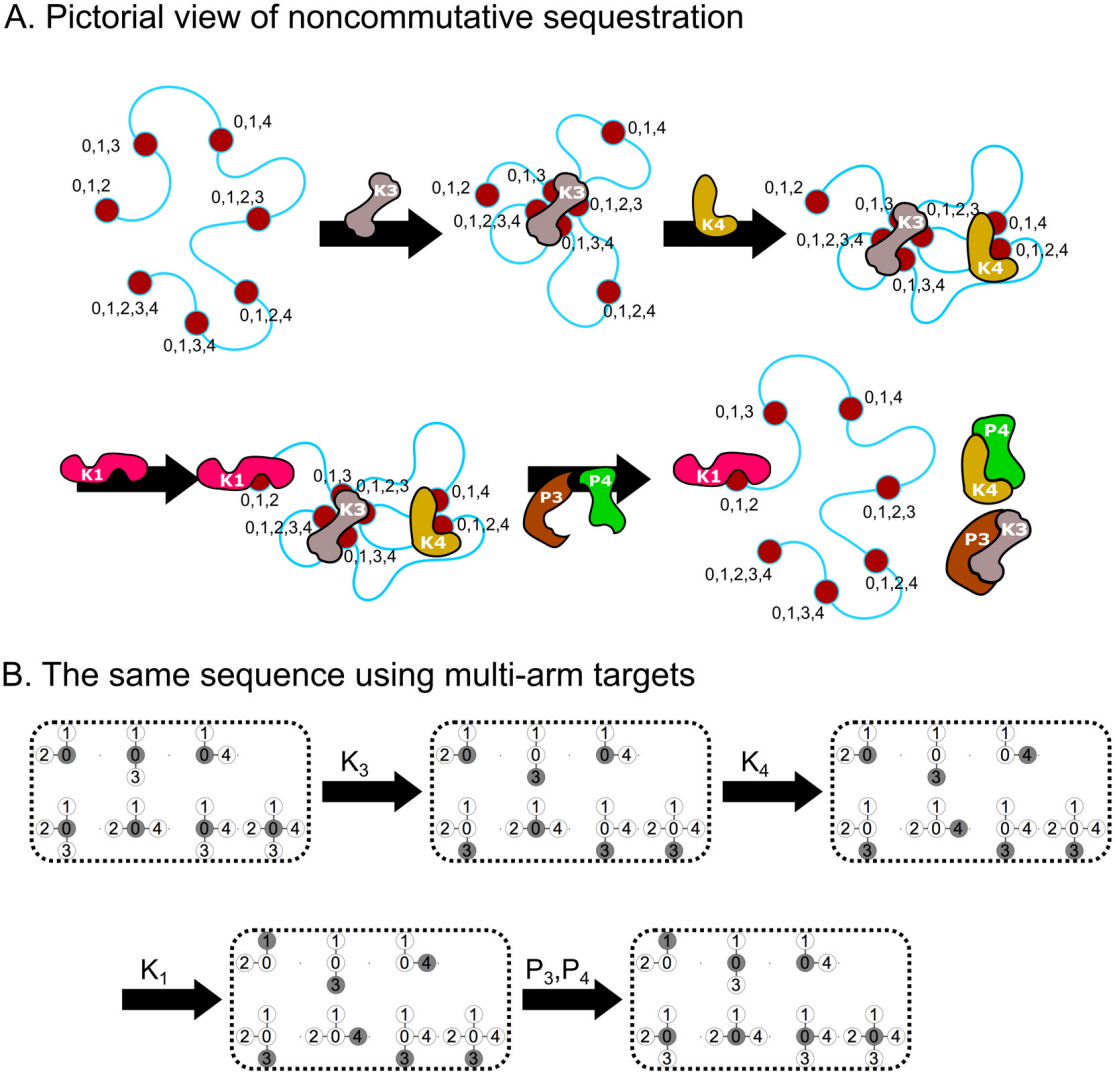
The sequestration network is a noncommutative model of gene regulation by chromosome folding. **(A)** A sequence of moves *K*_3_ *K*_4_ *K*_1_ *P*_3_ *P*_4_ on a hypothetical chromosome with *K* and *P* actions represented as DNA-binding factors and *K*_1_ playing the role of RNAP. Red circles correspond to genes and numbers correspond to allowed binding partners. **(B)** The same sequence in **A** represented as a collection of targets with up to *n* = 4 arms. For example, the target {0, 1, 2} corresponds to the gene locus with states 1 and 2 in **A**. The filled circle represents the current state.

To study the effect of accessibility and silencing on activating specific subsets of genes, we constructed the following sequestration model. In addition to the OFF state 0 and the ON state 1, each target/gene is endowed with additional orthogonal states 2 to *n* (allowing for a total of 2^*n* − 1^ − 1 genes). If RNA polymerase (RNAP) is associated with *K*_1_, what genes can be independently activated? In this model (Fig 5) a regulator *K*_*i*_ promotes targets in the 0 state to state *i*, and *P*_*i*_ returns targets in state *i* to 0. Any target in state *i* is protected from regulators other than *P*_*i*_. As an example of gene regulation on a three-dimensional chromosome (Fig 5A), the sequence *K*_3_ *K*_4_ *K*_1_ *P*_3_ *P*_4_ first clusters all genes having a 3 in a repressive compartment, and then the remaining genes having a 4 in another repressive compartment. The net effect is that RNAP can only act on the gene represented by {1, 2}. We represent this abstractly as a configuration vector of *k*-armed targets (Fig 5B), where each entry corresponds to the state {0, 1, …, *n*} of a gene able to access *k* ≤ *n* of the states (see below for a mathematical description of the model). Therefore, protected states in the sequestration model allow genes to be transcribed specifically in a well-mixed environment.

We derived (see below) that the number of reachable configurations scales with the number of regulator pairs *n* as

fn=22n-1-1-∑m=2n-1n-1m2∑k=3mmk-1-12∑k=3mn-1k-1-mk-1. (7)

**Fig 6:**
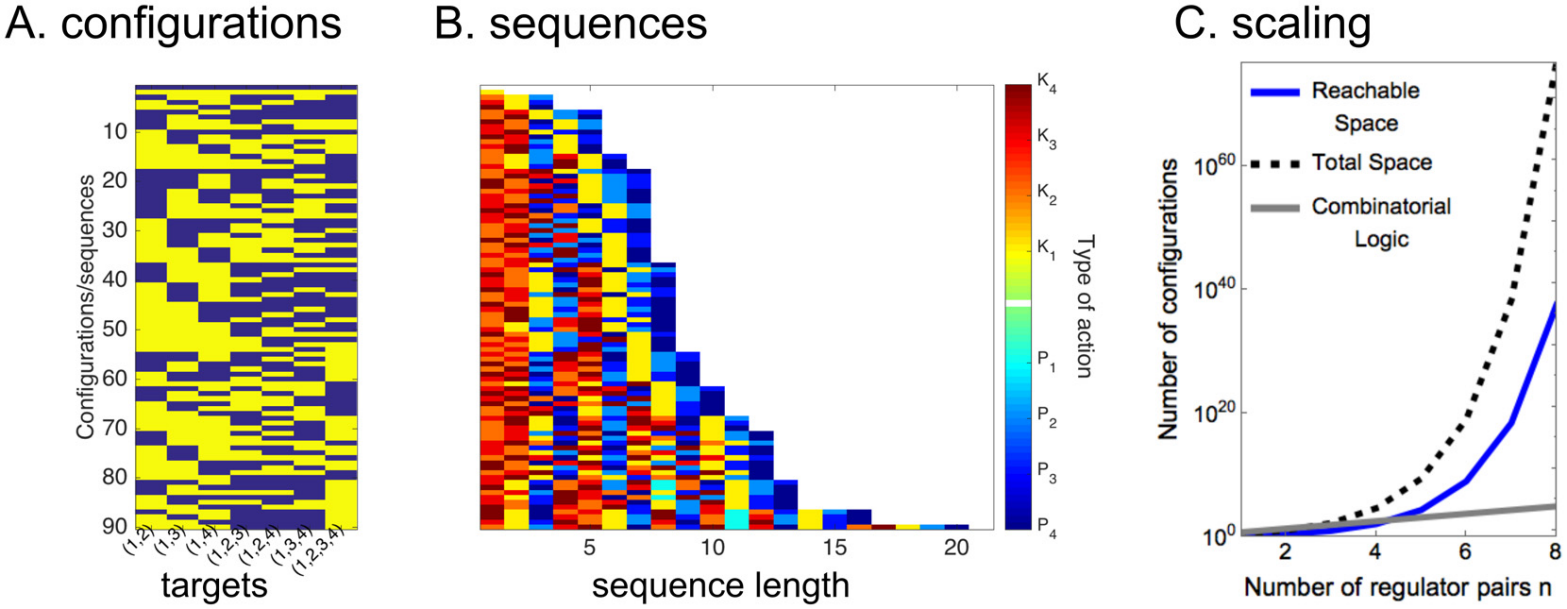
Scaling in the sequestration model is super-exponential. **(A)** A plot of all the allowed configurations of a set of targets of *n* = 4 regulator pairs in the sequestration model. Yellow represents targets that are ON, and blue those that are OFF. **(B)** A list of the sequences generating the corresponding states in **A**. *K* actions are shown in the red spectrum, and *P* in the blue. **(C)** A logarithmic plot of the scaling in the sequestration model. The total space is the 2^2^*n* − 1^ − 1^, the reachable space is calculated from Eq (7), and the combinatorial model is 2^2*n*^.

For *n* = 1, 2, 3, 4, 5, 6, this formula gives *f*(*n*) = 1, 2, 7, 89, 16897, 780304385 (Fig 6). We also relaxed the constraint that all genes have a 1 state (allowing for a total of 2^*n*^ − 1 genes) and found that the number of configurations scales as *c*_*n*_ = 2, 7, 94, 37701 with *n* = 1, 2, 3, 4. The full model does not have an analytical solution, but it does have upper and lower bounds related to Eq (7) (Materials and Methods Section 7, S3 Fig).

Combinatorial scaling laws of this sort are not uncommon [44, 64, 65]. Edwards and Glass [64] saw an explosion in the number of states when studying trajectories on *n*-cubes, and Green and Rees [65] saw a super-exponential jump when enumerating certain types of nonrepeating sequences on *n* letters. Furthermore, a similar small number (four) of factors are necessary and sufficient to reprogram fibroblasts to stem cells [66]. Together, these results indicate that sequences can far exceed the 2^*n*^ limit set by combinatorial regulation, and that only a few regulators are necessary to make large changes in the configuration of a cell.

### Regulators act on the configuration vector in the sequestration model

The sequestration network with *n* regulator pairs (referred to as the *n-network*) is described using the 1 × 2^*n*^ − 1 *configuration vector* **x**. This is a simpler description than the connectivity matrix because a target affected by *K*_*i*_ is necessarily affected by *P*_*i*_. The entries of **x** are the states of each target *g* able to be controlled by *k* ≤ *n* of the regulator pairs. Each target *g* is is a list {0, *i*_1_, …, *i*_*k*_} of the *k* regulators to which it responds. Because of their radial appearance, such targets are said to have *k* *arms* (see Fig 5B).

The regulators act on **x** according to the rules

$$\begin{array}{cc} K_{i}\left(x_{g}\right) & =\left\{\begin{array}{cc} x_{g}+i & \;\text{if}\;i\in \;g\;\text{and}\;x_{g}=0\\ x_{g} & \;\text{else} \end{array}\right.\\ P_{i}\left(x_{g}\right) & =\left\{\begin{array}{cc} 0 & \;\text{if}\;i\in \;g\;\text{and}\;x_{g}=i\\ x_{g} & \;\text{else.} \end{array}\right. \end{array}$$ (8)

Eq (8) guarantees that the regulators are *orthogonal* in the sense that a target in state *j* is protected from *K*_*i*_ and *P*_*i*_ if *i* ≠ *j*; and also *idempotent* in that $K_{i}^{2}=K_{i}$. Furthermore, sequences of regulators are noncommutative unless the only actions are *P*’s. This is a consequence of the fact that *P*’s put all affected targets into the 0 state. Although these rules are different from the ratchet model, a formulation exists that generalizes the *K*’s and *P*’s to matrix operators consistent with both models (Materials and Methods Section 9).

If **x** is restricted to the 2^*n* − 1^ − 1 targets all able to be regulated by *K*_1_ and at least one other *K*, the network is said to be *reduced*; otherwise we say **x** is *full*. This distinction was used in Fig 5.

A *one-coloring* is a configuration of **x** that uses only one of the states and 0. For example, the configuration **x** = (1, 0, 0, 1, 1, 0, 0) in the full *n* = 3-network is a one-coloring of 1; so is the reduced network formed by (**x**_4_, **x**_5_, **x**_7_) = (1, 1, 0). This concept is easily extended to *k* > 1-colorings. One-colorings are particularly important because they resemble the ON/OFF configurations of genes in an RNA-seq experiment, and we would like to know how many such configurations can be reached.

### A simple counting argument for the connected one-colorings illustrates super-exponential scaling in the sequestration model

As in the ratchet model, finding the accessible states of the sequestration network amounts finding restricted patterns in **x**. We determined that the restricted one-colorings are those that violate a property referred to as *connectivity* (Materials and Methods Section 7). A configuration of **x** is said to be connected if all *k* > 3-arm targets $g_{i}^{\left(k\right)}=\{0,i_{1},\ldots ,i_{k}\}$ match the state of at least one of *k* of the 2-arm targets {0, *i*_1_, *i*_2_}, …, {0, *i*_*k* − 1_, *i*_*k*_} sharing the indices *i*. If the network is reduced, no *k*-arm target may be in the 1 state when all of 2-arm targets with which it overlaps (i.e. shares an index other than 1) are in the 0 state. This restricts the one-colorings and suggests a method to determine the scaling law for the model in Fig 5.

As an example, in the *n* = 4 network on the reduced set of 2^3^ − 1 targets illustrated in Fig 5, {0, 1, 3} and {0, 1, 4} both being 0 constrains {0, 1, 3, 4} to be 0 as well. Furthermore, even though {0, 1, 2} is in the 1 state, {0, 1, 2, 4} and {0, 1, 2, 3, 4} may be 0. It is only the two-arm targets that constrain the possible configurations: for example, the longer sequence *K*_2_ *K*_4_ *P*_2_ *K*_3_ *K*_2_ *P*_4_ *K*_1_ *P*_3_ *K*_4_ *P*_1_ *K*_3_ *P*_4_ *K*_1_ *P*_2_ *P*_3_ obtains the state **x** = (0, 0, 1, 0, 0, 0, 1) in which only the targets {1, 4} and {0, 1, 2, 3, 4} are ON, showing that {0, 1, 2, 3, 4} need not be in the same state as {0, 1, 2, 3}, {0, 1, 2, 4}, or {0, 1, 3, 4}. In Fig 6A and 6B we show the allowed states and the sequences that generate them for *n* = 4; there are 90 out of a possible 2^2^4 − 1^ − 1^ = 128 configurations.

There are 2^2^*n* − 1^ − 1^ one-colorings on 2^*n* − 1^ − 1 targets. How many of these violate the connectivity rule? Suppose there are *m* 0’s among the 2-arm targets. If *m* = 1, then (mk-1)=(1k-1)=0 of the *k* ≥ 3-arm targets are constrained to be 0, as there is always another 2-arm target (in the 1 state) that each *k*-arm target can match. If *m* > 1 and *m* − 1 < *k*, however, then (mk-1)>0, so (mk-1) *k*-arm targets whose states {*i*_1_, …, *i*_*k* − 1_} are completely contained within the set of 2-arm targets {0, 1, *j*_1_}, …, {0, 1, *j*_*m*_} must be 0. Hence in any violation of the connectivity rules at least one of ∑k=3m(mk-1) *k*-arm targets will be in the 1 state and the remaining ∑k=3m(n-1k-1)-(mk-1) *k*-arm targets will be 0 or 1. Furthermore, there are (n-1m) ways of specifying *m* 0’s, so the total number of violations is

∑m=2n-1n-1m2∑k=3mmk-1-12∑k=3mn-1k-1-mk-1. (9)

Subtraction from 2^*n* − 1^ − 1 gives Eq (7).

### The ratchet and sequestration networks divide the configuration space into orbits

Until now we have considered the reachable space of a single group of targets each starting in 0. An ensemble of networks could each start with their targets in some arbitrary state, and when a sequence is applied to the ensemble the different networks will in general span different configurations. Determining the number of *orbits* (defined precisely in Materials and Methods Section 8) within the set of possible configurations tells us how many networks can be controlled in parallel.

Enumerating the reachable space for both the ratchet and sequestration networks involved finding configurations that violated at least one rule. If two configurations have distinct violations, then there is no way they can communicate using the regulators. Therefore, the different orbits are the groups of configurations having the same forbidden patterns. It is possible that a violation could be alleviated by an action that changes the state of an offending target, so we require that each orbit be immune to a subset of the regulators. This could be achieved in biological networks by locking targets in protective chromatin states or by shutting down certain cellular receptors.

**Fig 7:**
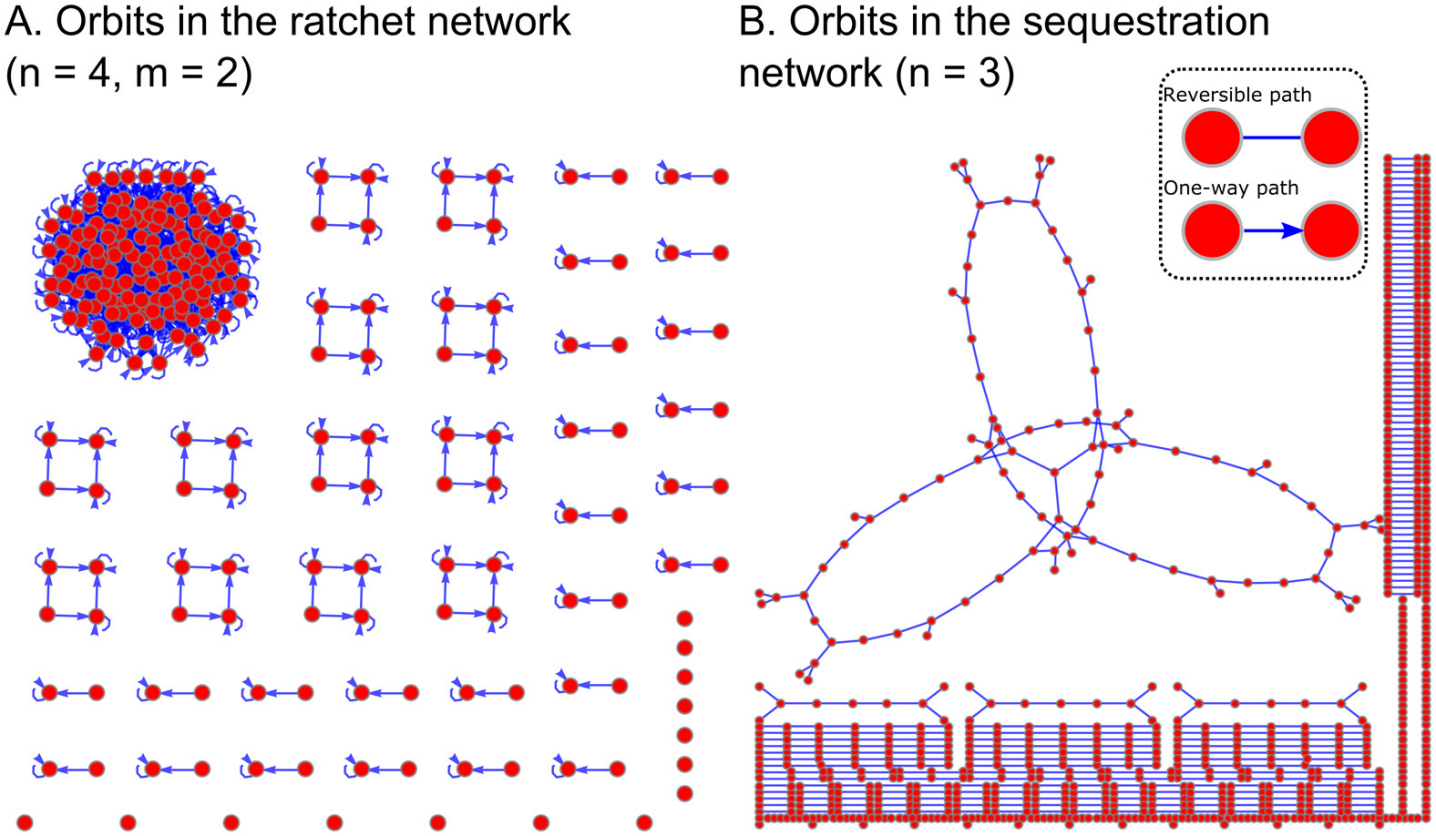
Noncommutative models induce orbits in the configuration space. Graphical representation of the orbits in **(A)** the *n* = 4, *m* = 2 ratchet network and **(B)** the full *n* = 3 sequestration network. Configurations are indicated by red circles, and those accessible to each other are connected with blue lines. Arrows in (A) indicate whether a path is irreversible.

We determined a recursive formula for the number of orbits in the ratchet network for an arbitrary *n*, *m* (Materials and Methods Section 8). In Fig 7A we plot the orbits for the *n* = 4, *m* = 2 case. There is one large component of size $B_{n}^{-m}$ and several smaller orbits of size $B_{i}^{-j}$ with *i* ≤ *n* and *j* ≤ *m*. There are only a handful of singleton orbits in Fig 7A, but the number of isolated states dominates the space as *n*, *m* increase.

We were unable to find a similar solution for the sequestration network because we lack a general solution for the number of states in the main orbit. However, Fig 7B shows the computationally discovered orbits for the full network on 2^*n*^ − 1 targets. A nontrivial feature is that there are orbits which use all pairs of regulators, but which do not communicate with the main orbit. For example, the sequence *K*_2_ *K*_3_ from **x** = (1, 0, 0, 0, 0, 0, 1) reaches the same configuration as the sequence *K*_1_ starting from **x** = (0, 2, 3, 2, 2, 3, 0); these configurations are part of the same orbit because both violate the connectivity rule between **x**_7_ = {0, 1, 2, 3} and the 2-arm targets **x**_4_, **x**_5_, and **x**_6_.

Another observation is that some pathways cannot be reversed by a legal action in the ratchet network orbits (indicated by a directed arrow in Fig 7), whereas there always exists a reversible path between configurations in the sequestration network orbits (no arrowheads). It can be proved that this is true in general for the sequestration network (Materials and Methods Section 8). This feature permits orbits to be found computationally by looking for reversible one-step paths in the entire configuration space.

The orbits are one explanation for the phenomenon the same signal can cause cells to behave differently [38]. More generally, the orbits demonstrate an intriguing symmetry between the targets responding to a restricted subset of the regulators on one hand, and the orbits restricted to the same subset on the other.

## Discussion

In this paper we first show how noncommutative, sequential logic can relieve information bottlenecks in multilayer networks. Bottlenecks in combinatorial logic may occur whenever a downstream layer has fewer elements than the layer upstream, which poses the problem of how networks process complex signals without loss of information. Noncommutative solutions such as the ratchet and sequestration models, in which the number of configurations scales super-exponentially in the number of regulators (Eqs (5) and (7)), permit longer, more complex messages to reach the targets via information “pulses.” These pulses encode a large diversity of signals into configurations of the targets that would otherwise be lost using combinatorial logic.

Noncommutativity has long been recognized as a central concept in control theory, because it allows systems with few controllers to explore a broader configuration space. For example, one generates *z* rotations in 3D by **R**_ − *x*_ **R**_*y*_ **R**_*x*_, so control over *z* is generated by a pulse sequence of rotations in *x* and *y*, as in airplane control where roll and pitch generate yaw [67]. Infinitesimal motions in the form of generating matrices are translated into flows in a vector space by exponentiation. Because matrix multiplication is noncommutative, composition of flows is not simply the addition of generators, but rather a higher order polynomial of commutators of the generators given by the Baker-Campbell-Hausdorff formula [68]. Noncommutativity also appears in experimental physical chemistry where pulse sequences can prepare spin systems in nontrivial population configurations [69]. A formal description of these phenomena is based on the Heisenberg picture of quantum mechanics, wherein evolution of a system of many variables is given by a differential equation involving the commutator of a Hamiltonian operator.

The significance of noncommutative control for systems biology is that it becomes possible to independently control targets that would otherwise be activated by the same promiscuous regulator. In this paper, we argue that noncommutative sequences permit control over new directions in gene expression space, allowing more specific sets of targets to be controlled. Several studies have shown that TFs that can bind genes in one tissue type are in fact precluded from binding the same genes in another [70, 71]. The C. elegans TF LIN35 fails to bind targets in the germline that it binds in the intestine [71], and the SMARCA4 complex in mouse binds enhancer elements in heart, limb, and brain tissue in a tissue-specific manner [70]. One hypothetical explanation for these observations, based on the sequestration model, is that cell-type specific gene expression is the result of noncommutative sequences like *K*_1_ *K*_2_ and *K*_2_ *K*_1_ that silence certain promoters. The three-dimensional structure of the genome is a likely setting for this type of regulation.

Gene regulation is known to take place in three-dimensions, as observations of DNA looping [72], nonrandom chromosome packing [73], and clustered transcription factories [74] have shown. However, the factors that affect chromosome structure are non-specific. One such factor is the ubiquitous zinc finger protein CCCTC binding factor (CTCF) [75], which functions as both an activator of transcription by bringing enhancers and promoters together [76, 77] and as a repressor by insulating genes [78, 79]. Epigenetic modifications, such as histone methylation and acetylation [80–82], also affect three-dimensional structure. In addition, DNA looping was observed in the context of allelic exclusion during B- and T-cell lineage specification where individual alleles were recruited to heterochromatic regions while the other underwent recombination [33, 34]. Consequently, the sequestration model predicts that temporal permutations of a small set of chromatin modifying factors could specify a large number of potential chromosomal conformations and lead to different expression states and corresponding cell fate decisions.

New technologies such RNA-seq and ChIP-seq can be used to test the predictions of the noncommutativity hypothesis at the genome level. Epigenetic drugs such as azacytidine and trichostatin A inhibit DNA methylation [83] and histone deacetylation [84], respectively, and have been shown to cause global changes in gene expression alone and in combination [83, 85]. The sequestration hypothesis predicts that perturbations to the three-dimensional structure of the chromosome are noncommutative, so distinct gene expression states may be reached by permuting the order in which epigenetic drugs are applied. While the sequestration model may underlie chromosome folding, the ratchet model could form the basis of phosphorylation networks. For example, mass spectrometry studies have revealed complex phosphorylation patterns [86, 87], though the number of kinases and phosphatases is comparatively small and the networks are highly interconnected [10, 11]. As phosphoproteins are the mediator of extracellular signals, ordered disruption of signaling pathways could also lead to distinct gene expression configurations.

Analogously, the ratchet model may aid in the specification of distinct neural activity patterns, owing to the fact that connections between the different hippocampal layers overlap [12, 88]. While superficial neurons can be activated in response to spatial cues, deeper layers can be selectively activated by time sequences of inputs [40, 41, 89]. These results suggest the hypothesis that neural networks may be noncommutative. In particular, experimental support exists for the role of the dentate gyrus in pattern separation and orthogonalization by way of ensuring that even quite similar memory representations use distinct subsets of neurons [90, 91]. The ratchet model, by ordering inputs in time, is one way of reaching these specific subsets if the number of input neurons is smaller than the number of targets neurons. Memories share many common elements, including shape, color, smell, and sound, which poses problems for recall. We hypothesize that older, “fuzzier” memories could be those relegated to very long ratchet sequences. According to this hypothesis, memories are not forgotten, but are instead increasingly difficult to access, and memories that are not consolidated are those that never formed a unique ratchet sequence.

Beyond resolving bottlenecks and generating specificity, noncommutative actions offer a new interpretation of how cell fate decisions and other stepwise processes occur on abstract regulatory landscapes. The classical Waddington landscape view of development holds that cells decay to attractor configurations representing terminal outcomes [92]; this is consistent with a boolean network with many variables *X* converging to a fixed point [93]. In a static landscape, the final outcome is determined *a priori* by the nearest energy minimum. What then determines the initial configuration? In organisms such as Drosophila, maternal patterning of the embryo may account for this initial bias [94]; but in other organisms that employ mechanisms like multilineage priming [82, 95], it is not clear that every cell fate decision is made at the beginning.

**Fig 8:**
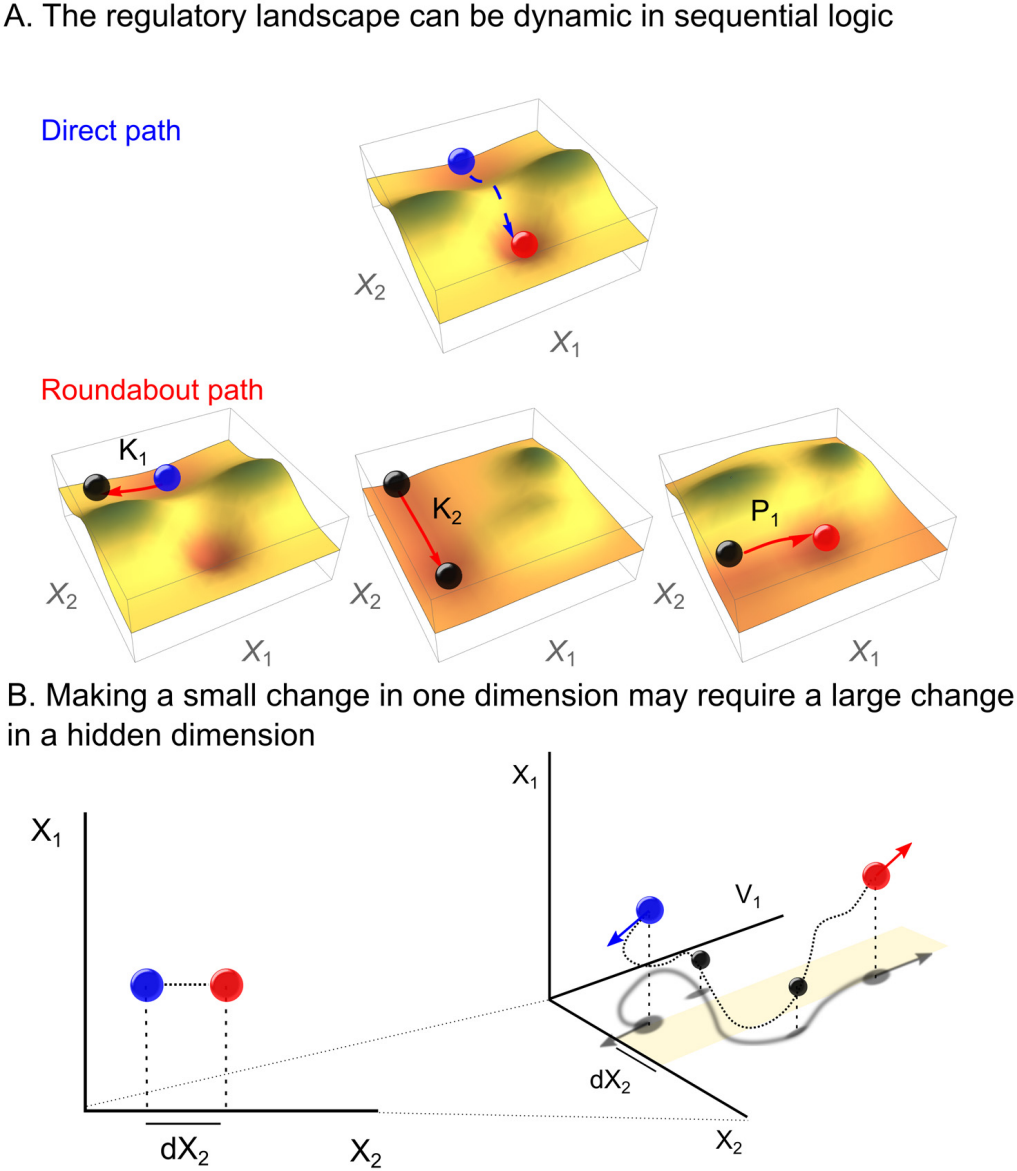
Sequential logic on regulatory landscapes. **(A)** The regulatory landscape for the 2-mRNA system *X*_1_, *X*_2_ for two hypothetical paths with configurations represented by balls. It is difficult to directly increase *X*_2_ because of a potential barrier (top). In the roundabout path (bottom), visiting two intermediate configurations via *K*_1_ *K*_2_ *P*_1_ results in an altered regulatory landscape. **(B)** The initial and final configurations in (A) projected onto (*X*_1_, *X*_2_) space (left) and (*X*_1_, *X*_2_, *V*_1_) space (right). The regulators affect not only *X*_1_ and *X*_2_, but also an additional variable, denoted *V*_1_, that alters the landscape of *X*_1_ and *X*_2_. The arrows indicate the instantaneous direction of the trajectory.

Sequential logic allows cells to reach their final fate on a dynamic landscape. In the system of Fig 8A (top), for example, it is not possible for cells in the blue configuration to transition to the red fate by increasing *X*_2_, because this involves an uphill climb. However, the regulators of genetic networks may also affect the landscape directly. This is seen in Fig 8A (bottom) where the sequence *K*_1_ *K*_2_ *P*_1_ changes the landscape in such a way that the overall cost of reaching the same endpoint is much lower than the direct path (Fig 8A, top). This can be understood as the effect of regulators acting on additional variables *V*, which modulates the landscape in *X* space. For example, TFs can recruit chromatin regulators that modify global three-dimensional chromosome structure and future TF accessibility [74, 76, 96, 97], or kinases can sequester substrates in the nucleus to prevent their subsequent activation [53, 54]. Because sequential logic acts on the *V*’s as well as the *X*’s, changes that appear to be small in one dimension (Fig 8B, left) actually involve large excursions in the full space (Fig 8B, right). As a consequence, in noncommutative regulation, the landscape changes and cells can take on fates that were not accessible at the beginning.

Previous theoretical models have explored dynamic regulatory landscapes in the form of bifurcations [98, 99]. In these models, a set of kinetic parameters determines the positions of minima and maxima in the landscape. However, the noncommutative model advanced here is fundamentally different, in that using the regulators to move through *X* changes the landscape directly. This could happen, for example, if acting on *X*_1_ with *K*_1_ hides it from the effect of *K*_2_. Uncoupling of targets in this way may underlie the distinct effects of signals like FGF at different stages of development [35–38]. It will be interesting to explore time series data for hints that some genes pulse ON and OFF in order to protect their promoters from the actions of promiscuous regulators.

Multistep processes other than development can benefit from the type of noncommutative regulation highlighted in Fig 8. What seems like an intractable problem at the start becomes much more feasible if one realizes that the effects of actions change with time and context. This intuition is why thinking in terms of commutators [*A*, *B*] = *AB* − *BA* can make complex problems more soluble: the desired effect is often what is leftover after performing and undoing a sequence of actions. Several examples illustrate this concept.

With its increased capacity for generating diversity, sequential logic is likely to be used in evolution. A recent theoretical example in social bacteria demonstrated that in evolving a new quorum sensing receptor-ligand pair, adding new receptors prior to ligands is preferred over the opposite path [45]. An analysis of the stability and catalytic activity of a family of bacterial *β*-lactamase mutants showed that the ability to evolve new substrate specificity is contingent on mutations that first stabilize the protein active site [46, 100]. Finally, biological networks evolve the same functions in different orders, but the order in which these functions arise dictates which other genotypes can be reached by neutral mutations [44]. These results suggest that permuted sequences of mutation events may have different fitness costs. With extensive artificial evolution experiments underway in protein engineering [100] and bacterial mutation accumulation [47], coupled with progress in sequencing technologies, it will be possible to test this hypothesis by permuting the conditions that promote mutation.

Sequential logic can also be applied in synthetic biology to build circuits with memory [43, 101–103]. In general, the toolkit that permits up- and downregulation of genes is small, with a few staples like Lac, Tet, and Ara [104]. Significant effort has been put into generating logic gate (AND/OR) promoters [30]. To further expand the toolkit, it has been proposed that more orthogonal regulators be developed [105]. We suggest that sequential logic may be a more promising strategy to scale up the number of targets that can be independently controlled by permuting in time a small number of controllers.

More broadly, sequential logic can be used to accomplish experimental goals not possible in single-step approaches. For example, in multiplexing mRNA detection in single cells, we previously used a sequential hybridization scheme that permits the number of barcodes to exponentially [106], whereas combinatorial schemes can only specify approximately 30 barcodes. We expect many single-cell experiments to benefit from a sequential strategy in which detours facilitate achievement of the main goal with high efficiency.

Finally, our results connect outside of biology to strategic planning in social, political, and economic arenas. Anyone familiar with negotiating knows about the limitations inherent in trying to make interconnected groups of people move in specific directions, especially when the actions affect all participants at once. Multiparty negotiations and tournaments may benefit from time-ordered strategies in which enemies temporarily team up, or fringe interest groups are transiently pacified. Indeed, a conclusion from the sequestration model is that the most highly regulated targets need to be protected prior to satisfying the ones with fewer connections. Determining whether this prediction is borne out in congressional and international negotiations, for example, is an interesting question for political science. Evidence for noncommutative effects in games exists in that the initial seeding in a tournament can bias its outcome [107], and that long-term goals change players’ strategies in in the repeated prisoner’s dilemma [108]. In conclusion, the direct path to an outcome in a networks with many interacting parts may have many unintended and prohibitively expensive consequences. A multi-step strategy may achieve the same outcome with minimal cost and side effects.

## Materials and Methods

### 1. The connectivity matrix with multiple targets

In this section we determine how many targets are controlled by the same regulators in the connectivity matrix **A**. Then we extend **A** to more than 2 dimensions.

If *l*_*n*_ = *l*_*m*_ = 1 it is clear that each **A**_*i*,*j*_ corresponds to a single target and that each target appears only once. In general, however, a target can appear in multiple entries of **A** (cf. Eq (6)). To see this, consider the bipartite graph formed by all the targets and all the *K*’s, but none of the *P*’s. The handshaking lemma from graph theory [59] says that the total number of edges is one half the sum of the degrees of each vertex, which is either *l*_*n*_ for a target or some number *p*_*n*_ for a *K* regulator. There are *Nl*_*n*_ total edges, so we find $\frac{1}{2}(Nl_{n}+np_{n})=Nl_{n}$ or $p_{n}=\frac{N}{n}l_{n}$ for the number of links coming from each *K*. Similarly, the number of links emanating from each *P* is $p_{m}=\frac{N}{m}l_{m}$. In terms of the connectivity matrix, *p*_*n*_ and *p*_*m*_ correspond to the number of unique targets in each row and column, respectively.

Because *K*_1_ connects to a fraction $\frac{p_{n}}{N}$ of the targets, it follows that *K*_1_ and *P*_1_ together connect to a fraction $\frac{p_{n}p_{m}}{N^{2}}$ of the targets. Therefore, the total number of targets connecting to *K*_1_ and *P*_1_ is $M=N(\frac{p_{n}p_{m}}{N^{2}})=\frac{p_{n}p_{m}}{N}$. Another way to see this is to consider one target in the intersection of *K*_1_ and *P*_1_. This one target uses up one of each of the regulators and one unit of connectivity, leaving a total of M=(n-1ln-1)(m-1lm-1) ways to connect other targets to the same pair of regulators. It is easily verified that these two formulations for the number of targets per matrix entry *M* are equivalent. This illustrates that there is not simply a one-to-one correspondence between the entries of **A** and the targets.

There was nothing special about the labels *K* and *P* in the above paragraphs. Thus, the connectivity matrix can easily be extended to a *u*-dimensional *connectivity tensor* where *u* is the number of pools of regulators. Each pool has *n*_*i*_ regulators connecting to *l*_*ni*_ targets, and each target connects to $p_{ni}=\frac{N}{n_{i}}l_{ni}$ regulators of pool *i*, ∀*i* ∈ {1, …, *u*}. The total number of targets and the total number of targets per entry are extensions of the *u* = 2 case, giving

N=∏i=1unilni (10)

distinct targets and

M=∏i=1upniNu-1=∏i=1uni-1lni-1 (11)

targets controlled by one factor from each of the *u* pools. S1A Fig shows an example network with *u* = 3 pools.

### 2. Counting configurations in combinatorial networks using the connectivity matrix

The number of configurations in combinatorial logic is the number of ways that *N* targets can each be bound by exactly *u* regulators, where each regulator comes from a different pool. In the main text we analyzed the case *u* = 1 and *l*_*n*_ = 1. Here we extend those results to arbitrary *u* and *l*_*n*_.

First consider the case *u* = 2, corresponding to a pool of *K*’s and a pool of *P*’s. Whereas in the ratchet model, *K*_*i*_ and *P*_*j*_ acted separately on the entries of **A**, in combinatorial logic the pair (*K*_*i*_, *P*_*j*_) is needed to switch **A**_*i*, *j*_ from 0 to 1. Many such pairs may be active at any one time. We write this formally as

$$\begin{array}{c} \left(\left\{K\right\},\left\{P\right\}\right)\left[A_{i,j}\right]=\left\{\begin{array}{cc} 1 & \;\text{if}\;K_{i}\in \left\{K\right\}\;\text{and}\;P_{j}\in \left\{P\right\}\\ 0 & \;\text{else,} \end{array}\right. \end{array}$$ (12)

where {*K*} denotes a subset of the *K*’s. The notation (⋅, ⋅) means that a combination of factors acts on the target, instead of just a single factor.

If *l*_*n*_ = *l*_*m*_ = 1 there are (2^*n*^ − 1)(2^*m*^ − 1) + 1 ways to pick at least one of *n* *K*’s and one of *m* *P*’s, plus one way to pick nothing. If *l*_*m*_ = 1 and *l*_*n*_ > 1, then for a certain number *α* ≤ *n* of the *K*’s, any subset containing *α* or more *K*’s has the same effect as activating all *n* *K*’s at once. For example, in Eq (6), the action of ({*K*_1_, *K*_2_}, {*P*_1_, *P*_2_}) is sufficient to activate all targets in the *n* = *m* = 3, *l*_*n*_ = *l*_*m*_ = 2 network. To determine *α*, recall that there are *M* targets in each entry of the connectivity matrix **A**. Choosing *i* *K*’s means that the total number of targets is *M* × *i*, but a single column of **A** only contains *p*_*m*_ unique targets. Each target is connected to *l*_*n*_ *K*’s, so for a target in the intersection of *i* *K*’s and a single *P*, there are *l*_*n*_ − *i* spots left over to choose *n* − *i* *K*’s and *l*_*m*_ − 1 spots left over to choose *m* − 1 *P*’s, or (n-iln-i)(m-1lm-1) ways total. Using the principle of inclusion-exclusion [59] this means that *α* is the smallest *i* such that

M×i-∑i′=2mini,ln-1i′ii′n-i′ln-i′m-1lm-1≥pm. (13)

By choosing *α* *K*’s, the number of unique targets in a column of **A** that can be turned ON is exactly the number represented in that column. Because all subsets with *α*, *α* + 1, …, *n* − 1 *K*’s are redundant, here are only (2n-1)-∑i=αn-1(ni) subsets of *K*’s that contribute to unique configurations, leaving a total of [(2n-1)-∑i=αn-1(ni)](2m-1)+1 unique configurations.

If the *P*’s also have redundant connections, the result generalizes to

**Theorem 1** *The number of configurations in combinatorial logic with parameters n, m, l_n_, l_m_, and u* = 2 *is*

2n-12m-1+1-∑i=αn-1ni2m-1-2n-1∑i=βm-1mi+∑i=αn-1ni∑i=βm-1mi, (14)

*where α (resp. β) is the smallest number of K’s (resp. P’s) having the same effect as all K’s (resp. P’s) at once.*

This result is obtained by counting all pairings of *K*’s and *P*’s, then subtracting those pairings that have a redundant effect. For example, any combination using *K*_3_ is redundant in the connectivity matrix of Eq (6). Finally, those pairings that were excluded twice are added back in.

This result generalizes to all *u* with slight modifications. Because one factor from each of *u* pools is now required, the combinatorial equation determining state of a target is

$$\begin{array}{c} \left({\left\{K\right\}}_{1},{\left\{K\right\}}_{2},\ldots ,{\left\{K\right\}}_{u}\right)\left[A_{i,j,\ldots ,k}\right]=\left\{\begin{array}{cc} 1 & \;\text{if}\;K_{1i}\in {\left\{K\right\}}_{1},K_{2j}\in {\left\{K\right\}}_{2},\ldots ,K_{uk}\in {\left\{K\right\}}_{u}\\ 0 & \;\text{else.} \end{array}\right. \end{array}$$ (15)

Here the double subscript *K*_*ik*_ indicates the *k*^th^ factor in the *i*^th^ pool. Determining *α*_*i*_ for each pool *i* of regulators requires finding the pool *j* ≠ *i* which maximizes the number *N*_*i*_ of targets controlled in two dimensions. If we choose *α*_*i*_ or more regulators in the *i*^th^ pool, then there is a redundancy in the *j*^th^ dimension, whereas any choice of fewer than *α*_*i*_ regulators activates fewer than *N*_*i*_ targets. Write Ni=maxj≠i{(nilni)(njlnj)} the total number of targets and $p_{nj}=\frac{N_{i}}{n_{j}}l_{nj}$ the number of targets in any column of the the equivalent *n*_*i*_ × *n*_*j*_ connectivity matrix regulated by pools *i* and *j*. It is easy to see that these parameters reduce to their previous definitions for *u* = 2. Now define Mi=(ni-1lni-1)(nj-1lnj-1) as the number of targets in each entry of the equivalent *n*_*i*_ × *n*_*j*_ connectivity matrix. As above, *α*_*i*_ is now the smallest *r* such that

Mi×r-∑r′=2minr,lni-1r′ni-r′lni-r′nj-1lnj-1≥pnj. (16)

Once *α*_*i*_ is determined for each pool *i*, the inclusion-exclusion sum can be extended using standard arguments [59]. Define by

Sk=∑σ∈uk∏i∈σ∑j=αini-1nij∏i∉σ2ni-1, (17)

where *σ* denotes all *k*-subsets of {1, …, *u*}. Then we have the final result

**Theorem 2** *The total number of configurations in combinatorial logic with u pools and parameters n_i_, l_ni_, i* ∈ {1, …, *u*} *is*

$$\begin{array}{c} S=1+\sum_{k=0}^{u}{\left(-1\right)}^{k}S^{\left(k\right)}. \end{array}$$ (18)

This result reduces to Theorem 1 when there are only *u* = 2 pools. At most there are $\prod_{i=1}^{u}(2^{n_{i}}-1)$ ways to specify at least one target, corresponding to the 0^th^-order term in Eq (18). Increasing the connectivity through the *l*_*ni*_ can only reduce the number of configurations. This behavior is shown in S1B Fig for the symmetric case that all the *n*_*i*_ and *l*_*ni*_ are equal. As *u* is increased the number of configurations increases dramatically, but the scaling is actually subexponential, i.e. less than 2^*N*^. Increasing connectivity through *l*_*ni*_ shifts the curves to the right.

### 3. Using the connectivity matrix to establish a one-to-one correspondence between the ratchet network and the lonesum matrices

To establish the correspondence between the reachable configurations of ratchet network (*l*_*n*_ = *l*_*m*_ = 1, *T* = 1) and the lonesum matrices, we must show (i) that **A** avoids the patterns $\left(\begin{array}{cc} 1 & 0\\ 0 & 1 \end{array}\right)$ and $\left(\begin{array}{cc} 0 & 1\\ 1 & 0 \end{array}\right)$ in any 2 × 2 sub-block, and (ii) that any lonesum matrix can be constructed from *K* and *P* actions. First observe that the value 1 in **A**_*i*, *j*_ indicates the last *K* affecting that index must have followed a *P*, whereas 0 indicates the last *P* must have followed a *K*. For the first restriction we have $\left(\begin{array}{cc} 1 & 0\\ 0 & 1 \end{array}\right)$ implies $\left(\begin{array}{cc} P_{1}\ldots K_{1} & K_{1}\ldots P_{2}\\ K_{2}\ldots P_{1} & P_{2}\ldots K_{2} \end{array}\right)$. This means *P*_2_ follows *K*_1_ follows *P*_1_ follows *K*_2_ follows *P*_2_, which is a contradiction, showing that this 2 × 2 block is unreachable. The other five unique 2 × 2 blocks are all reachable with elementary sequences. This establishes point (i) that the reachable configurations are a subset of the lonesum matrices.

To establish point (ii) that the lonesum matrices are a subset of the reachable configurations, we use an equivalent formulation of the lonesum matrices as *staircase matrices* composed of the rows *a*_*j*_ = (1, …, 1, 0, …, 0) with the last 1 appearing at position *i*_*j*_ subject to the constraint that *i*_*j*_ ≤ *i*_*j* − 1_ for all ∀*j* ∈ {2, …, *n*} [109]. It is easy to see that the pattern of ones resembles an inverted staircase. We show via induction that any staircase matrix can be constructed from *K* and *P* actions. The *n*^th^ row is obtained by the sequence *K*_*n*_ *P*_*i*_*n*_ + 1_⋯*P*_*m*_ which leaves 1’s at the first *i*_*n*_ indices and 0’s at the remainder. Now assume that the *k*^th^ row is obtained by the sequence *K*_*k*_ *P*_*i*_*k*_ + 1_⋯*P*_*m*_ without affecting any of the rows *n*, *n* − 1, …, *k* + 1. Then the sequence *K*_*k* − 1_ *P*_*i*_*k* − 1_ + 1_⋯*P*_*m*_ puts 1’s at the first *i*_*k* − 1_ indices of row *k* − 1. Because *i*_*k* − 1_ ≥ *i*_*k*_ ≥ ⋯ ≥ *i*_*n*_, none of the *P*_*i*_*k* − 1_ + 1_, …, *P*_*m*_ turn a 1 to a 0 in rows *n*, *n* − 1, …, *k* + 1, *k*. This proves the induction hypothesis and shows that the staircases matrices are a subset of the reachable configurations.

Together with the fact that the reachable configurations are a subset of the staircase matrices, this implies that the reachable configurations and the lonesum matrices are in fact the same set, and we have

**Theorem 3** *The number of reachable configurations in the* (*n*, *m*) *ratchet network with l_n_ = l_m_* = 1 *and threshold 1 scales as the poly-Bernoulli numbers* $B_{m}^{-n}=B_{n}^{-m}$.

### 4. Inductive proof that all binary ON/OFF configurations are reachable in the ratchet network with threshold greater than 1

With *T* = 2, only targets in state 2 are ON. Once a 0-1 configuration of **A** is obtained, however, it is a simple matter to convert it into an ON/OFF configuration by applying all the *K*’s. Here we use the fact that 1’s can be reached from above and below to prove the

**Theorem 4** *In the ratchet network represented by the matrix* **A** *with l_n_ = l_m_* = 1 *and threshold* *T* = 2, *all binary 0-1 configurations are reachable*.

*Proof*. We use an induction argument analogous to the proof of Theorem 3. Suppose that in row *n* a set of *r* ≤ *m* indices {*n*_*j*_} = {*n*_*j*_1__, …, *n*_*j*_*r*__} should be ON. First prepare every target in row *n* in the 1 state using *K*_*n*_, then use the sequence *K*_*n*_ *P*_*j*_*r* + 1__⋯*P*_*j*_*m*__ to obtain 2’s at {*n*_*j*_1__, …, *n*_*j*_*r*__} and 1’s at {*n*_*j*_*r* + 1__, …, *n*_*j*_*m*__}. Now assume that we can prepare rows *n*, *n* − 1, …, *k* + 1 in a similar 1-2 configuration with the rest of the matrix 0. We want to show that we can add row *k* to this set without affecting any of the previous rows. Assuming that a set of *s* ≤ *m* indices {*k*_*j*_1__, …, *k*_*j*_*s*__} should be ON, apply the sequence $P_{j_{1}}\ldots P_{j_{s}}K_{k}^{2}P_{j_{s+1}}\ldots P_{j_{m}}$ to obtain 2’s at {*k*_*j*_1__, …, *k*_*j*_*s*__} and 1’s at {*k*_*j*_*s* + 1__, …, *k*_*j*_*m*__}. Now, because {*P*_*j*_1__, …, *P*_*j*_*s*__}∪{*P*_*j*_*s* + 1__, …, *P*_*j*_*m*__} = {*P*_1_, …, *P*_*m*_}, all 2’s and 1’s in rows *n*, *n* − 1, …, *k* + 1 are now 1’s and 0’s, respectively. Applying the sequence *K*_*n*_ *K*_*n* − 1_⋯*K*_*k* + 1_ reestablishes the 1-2 configuration we had prior to fixing row *k* and leaves 0’s at rows 1, …, *k* − 1. Now that row *k* is also in the proper 1-2 configuration, we have proved the induction hypothesis. Once all rows in the proper 1-2 configuration, the sequence *P*_1_⋯*P*_*m*_ obtains the matrix in the 0-1 configuration. Since this procedure can be repeated for any collection of indices {{1_*j*_}, …, {*n*_*j*_}}, it follows that all binary 0-1 matrices are reachable.

### 5. A recursive formula for the number of non-redundant sequences in the ratchet network

When the connectivity parameters *l*_*n*_ and *l*_*m*_ exceed 1, certain sequences in the threshold 1 ratchet network become redundant. Our goals in this section are to (i) to characterize the redundant sequences by the number of *K*’s and *P*’s, and (ii) count the non-redundant sequences. This will obtain an upper bound on the number of configurations.

We want the shortest sequences that can activate or (deactivate) all targets; any sequences longer than this are redundant. To see why this is so, we need the concept of a *cycle*. We say that a target has gone through a cycle if has traversed the states 0, 1, 0 at some subsequent time points. We have the following lemma.

**Lemma 5** *Any sequence that takes all targets through a cycle is redundant*.

*Proof*. The final configuration of any sequence is represented by the positions of the 1’s and 0’s of the connectivity matrix. Recall that **A**_*i*,*j*_ = 0 if an only if all targets represented by **A**_*i*,*j*_ are OFF in the final configuration. Permute the rows and columns of **A** until it is in staircase form with *r* ≤ min(*n*, *m*) steps, where a step is a group of adjacent rows or columns having the same number of 1’s and 0’s. The steps partition the rows and columns of **A** into subsets of indices {*i*_1_, *i*_2_, …, *i*_*r*_} and {*j*_1_, *j*_2_, …, *j*_*r*_} where the *k*^th^ step is defined by 1’s at rows *i*_*k*_ to *i*_*k* + 1_ − 1 and 0’s at columns *j*_*k*_ to *j*_*k* + 1_ − 1. Then the sequence $\prod_{k=1}^{r}K_{i_{k}}\ldots K_{i_{k}-1}P_{j_{k}}\ldots P_{j_{k}-1}$ obtains the desired configuration of 1’s and 0’s. Being able to write a staircase matrix for the final configuration means that every target ON in the final configuration occurs only where there are 1’s in the matrix. These targets are never affected by a *P* in this procedure; they do not go through a cycle. Because any allowed configuration can be reached from this procedure, it follows that any sequence that uses a cycle is redundant.

Knowing that the non-redundant sequences must avoid cycles, it suffices to find the longest sequences that can be written before cycles appear.

**Lemma 6** *For each value of l_n_ (l_m_), the maximum number of K’s (P’s) that can be used before all targets are activated (deactivated) is n − l_n_ +* 1 *(m − l_m_)*.

*Proof*. A sequence that activates all targets has no intervening *P*’s. Recall that a single *K* activates at most $\frac{N}{n}l_{n}$ targets. Then, prior to the last *K* being used, the number of activated targets is $N-\frac{N}{n}l_{n}=\frac{N}{n}(n-l_{n})\leq \frac{N}{n}l_{n}(n-l_{n})$. This means there are at most *n* − *l*_*n*_ groups of targets controlled by different *K*’s. Thus, at most *n* − *l*_*n*_ *K*’s are used before the last *K* is used, and *n* − *l*_*n*_ + 1 *K*’s must be sufficient to activate the complete set. The maximum number of *P*’s that can be used is only *m* − *l*_*m*_ because we can think of every sequence starting in the zero configuration as having been preceded by a single *P*; this modification puts the *P*’s on equal footing with the *K*’s.

With this characterization of the non-redundant sequences our goal is to recursively eliminate sequences that use *n* − *l*_*n*_ + 1 *K*’s and *m* − *l*_*m*_ *P*’s. We first find the number of sequences that use up to *m* − *l*_*m*_ *P*’s, which forms the top row in each (*n*, *m*) block in S2 Fig. Then we use these values to recursively find the number of sequences using up to *n* − *l*_*n*_ + 1 *K*’s. The strategy is to subtract from the total number of sequences at a given (*l*_*n*_, *l*_*m*_) all those sequences using the forbidden number of regulators in order to get the new total.

Denote by $a_{n}^{m}$ the number of sequences using *m* *P*’s when the total number of *K*’s is *n*. If *m* = 1, then all $B_{1}^{-n}=2^{n}$ sequences (except for the empty sequence) use a *K* and none use a *P*. If *m* = 2, the maximum number of *P*’s that can be used is *m* − *l*_*m*_ = 1. Discarding the 2^*n*^ sequences with no *P*, the number of sequences using a single *P* is

$$\begin{array}{c} a_{n}^{1}=\frac{B_{2}^{-n}-2^{n}}{2}. \end{array}$$ (19)

Division by *m* = 2 is required to account for the fact that there are (m1)=m different ways of starting each sequence with a *P*, and we consider both of these equivalent. Having determined $a_{n}^{m}$, it is straightforward to determine $a_{n}^{m+1}$. Because there are *m* + 1 *P*’s to choose from, there are (m+1m)anm ways to write sequences with *m* *P*’s, (m+1m-1)anm-1 ways to write sequences with *m* − 1 *P*’s, …, (m+10)1 ways to write sequences with 0 *P*’s, the only remaining sequences are those with *m* + 1 *P*’s. Knowing that the total number of sequences is $B_{m}^{-n}$, this leaves

anm+1=Bm-n-2n-∑j=0mm+1janjm+1 (20)

total sequences using *m* + 1 *P*’s when the total number of *K*’s is *n*. Having determined this number, we can sum up all the sequences using *m* − *l*_*m*_ *P*’s to get the first row of the (*n*, *m*) block in S2 Fig. Denote by $c_{n}^{m}(l_{n},l_{m})$ the *l*_*m*_^th^ column and *l*_*n*_^th^ row of the (*n*, *m*) block. The column headers $c_{n}^{m}(1,l_{m})$ are given by

cnm1,lm=2n+∑j=0m-lmmjanj. (21)

We can determine the row entries for *l*_*n*_ > 1 in the same way that we determined the column headers, the only difference being that the total number of sequences is $c_{n}^{m}(1,l_{m})$, not $B_{m}^{-n}$ unless *l*_*m*_ = 1. Denote by $b_{m}^{n}(l_{m})$ the number of sequences using *n* *K*’s when the total number of *P*’s is *m* and the *P* connectivity is *l*_*m*_. For fixed *m*, *l*_*m*_ and *n* = 1, there are

bm1lm=2m-∑j=0lm-1mj, (22)

sequences, as all but the empty sequence use a single *K*. In complete analogy to Eq (20) we find there are

bmn+1lm=cnm1,lm-∑j=0n-ln+1n+1jbmjlm (23)

sequences using *n* + 1 *K*’s when the total number of *P*’s is *m*. Unlike in the equation for $a_{n}^{m}$, there is no division by *n* + 1 because all sequences starting with a different *K* are different. Finally, we can sum up all the sequences using *n* − *l*_*n*_ + 1 *K*’s to get the

**Theorem 7** *The number of minimal length sequences in the* (*n*, *m*, *l_n_*, *l_m_*) *ratchet network with threshold T* = 1 *using no more than n* − *l_n_* + 1 *K’s and m* − *l_m_ P’s is*

cnmln,lm=∑j=0n-ln+1njbmjlm. (24)

We used this formula to compute each entry in S2 Fig. Because of the complexity of this procedure, we checked it against a computer algorithm operating with the following steps. In step 1 find all $B_{m}^{-n}$ sequences in the *l*_*n*_ = *l*_*m*_ = 1 case. In step 2 increase the connectivity (*l*_*n*_ or *l*_*m*_) and find all sequences of a given length; group them by the configuration they generate. Some of these sequences will not appear in the list generated by step 1: for example, both *K*_1_ *K*_2_ and *K*_2_ *K*_1_ will be found in step 2. We are interested in index permutation e.g. 1 → 3, not letter permutation, so in step 3 delete all sequences in each length group not appearing in step 1. Repeat steps 1–3 with this new list of sequences until *l*_*n*_ = *n* − 1. This code, implemented in Matlab Version 2015b, gave exact agreement with Theorem 7.

### 6. Proof that the reachable configurations are equivalent to the connected one-colorings

We now show that rules restrict the reachable configurations of the sequestration model in the main text to the connected one-colorings of the reduced *n*-network.

**Theorem 8** *There is a one-to-one correspondence between the reachable configurations of the reduced n-network and the connected one-colorings*.

*Proof*. The converse direction, reachable implies connected, is easier to prove and will be discussed first. Assume that all configurations in the reduced *n*-network so far reached are connected. The next configuration will be reached by turning all 0’s to *i*’s or all *j*’s to 0’s by application of *K*_*i*_ or *P*_*j*_, respectively. The *k*-arm targets sharing state *i* with the 2-arm target {0, 1, *i*} are either in the same state as some other 2-arm target {0, 1, *i*′} or are in the 0 state. So application of *K*_*i*_ cannot change the connectivity of the configuration. Furthermore, a *k*-arm target can be in the *j* state only if the target {0, 1, *j*} is in the *j* state, so these targets will still be matched after application of *P*_*j*_. Thus, any configurations reachable from a reachable configuration must be connected.

The forward direction, connected implies reachable, is less trivial. In order to prove that all connected one-colorings in the *n*-network are reachable, we will use the strong form of mathematical induction. Assume the theorem holds for all networks up to *n* − 1. Embedded within the full *n*-network of 2^*n*^ − 1 targets is the reduced *n*-network on 2^*n* − 1^ targets. Within the reduced *n*-network is a set of 2^*n* − 2^ targets able to access {0, 1, 2} and all subsets (including Ø) of the integers {3, …, *n*}. Thus, we can substitute 2 → 1 as the ON state in this embedded network and all connected one-colorings (of 2) will be reachable. The same holds in general for all 2^*n* − *k*^ targets able to access {0, 1, *k*} and all subsets of the integers {*k* + 1, …, *n*}. In each of these embedded networks the substitution *k* → 1 as the ON state will enable us create any connected one-coloring.

Pick any connected one-coloring (of 1) in the *n*-network. Its *opposite* configuration is formed by the transformation at each target *g* of 1 → 0 and 0 → *k*_*min*_, where *k*_*min*_ = min{*k*∈*g*|**x**_pos({0, *j*, *k*})_ = 0} is the smallest index that *g* shares with a corresponding 2-arm target at position pos({0, *j*, *k*}) of **x** (possibly in the full network) currently in the 0 state. The opposite of a connected one-coloring is clearly connected, because all the connected 1’s are now 0, and all the 0’s are in the same state as the 2-arm target {0, *j*, *k*_*min*_}. If it is possible to reach the opposite configuration, then application of the sequence *K*_1_ *P*_2_…*P*_*n*_ yields the desired one-coloring of the *n*-network.

To show that the opposite configuration of the chosen one-coloring is indeed reachable, isolate the embedded networks one-by-one by application of the sequence *K*_*k*_ *K*_1_ *P*_*k*_ for *k* = 2, …, *n*, so that the targets in the *n* − *k* + 1-network are the only targets in the 0 state. By hypothesis, the connected one-colorings are reachable in all embedded networks which have at most *n* − *k* states besides 0, 1, and *k*. The opposite configuration in the *n*-network is composed of connected one-colorings (of *k*) in each embedded network; these are are reachable. Therefore, the one-coloring of the *n*-network is reachable via *K*_1_ *P*_2_…*P*_*n*_. This procedure holds for any one-coloring.

### 7. Lower and upper bounds for the full *n*-network

How many configurations are reachable in the full *n*-network? Let this number be *c*_*n*_. The following theorems derive lower and upper bounds for *c*_*n*_ in terms of the number of one-colorings.

**Theorem 9** *The formula* *f*(*n* + 1) *for the number of connected one-colorings in the reduced* *n* + 1-*network is a lower bound for* *c*_*n*_.

*Proof*. The full *n* + 1-network can be partitioned into a set of 2^*n*^ targets having a 1 and all subsets of {2, …, *n* + 1}, and 2^*n*^ − 1 targets that lack 1 but have all nonempty subsets of {2, …, *n* + 1}. The latter set of targets is an embedded full *n*-network, while the former is the reduced *n* + 1-network. All 2(*n* + 1) letters are needed to form the one-colorings in the reduced *n* + 1-network. Every one-coloring is finally obtained by applying some permutation of *K*_1_, *P*_2_, …, *P*_*n* + 1_ to a configuration that uses (at most) the states 2, …, *n* + 1 and 0, i.e. the full *n*-network. Because *K*_1_ and *P*_1_ do not affect the targets of the the embedded full *n*-network, there must be (at least) one sequence using only {*K*_2_, …, *K*_*n* + 1_} and {*P*_2_, …, *P*_*n* + 1_} that prepares the embedded full *n*-network in the aforementioned configuration, which means we may associate a one-coloring with (at least) one of the *c*_*n*_ sequences in the embedded full *n*-network. Therefore, multiple configurations in the full *n*-network may map to the same one-coloring in the reduced *n* + 1-network. Conversely, if two one-colorings are different, they are distinguishable by their configurations immediately preceding the final *K*_1_, *P*_2_, …, *P*_*n* + 1_ sequence, and must therefore map to different configurations in the full *n*-network. Together, these statements imply that the map from configurations in the full *n*-network to one-colorings in the reduced *n* + 1-network is many-to-one, but the map from one-colorings to configurations in the full *n*-network is one-to-one. Therefore, *f*(*n* + 1) ≤ *c*_*n*_.

**Theorem 10** *An upper bound on* *c*_*n*_ *is*

$$\begin{array}{c} nf\left(n\right)+n\left(n-1\right)\left(f\left(n\right)-1\right)f\left(n-1\right)+\cdots +n!\left(f\left(n\right)-1\right)\cdots \left(f\left(2\right)-1\right)f\left(1\right)+1\\ \;=\sum_{k=1}^{n}{\left(n\right)}_{k}\left\{\prod_{j=n-k+2}^{n}\left(f\left(j\right)-1\right)\right\}f\left(n-k+1\right)+1. \end{array}$$ (25)

*where* (*n*)_*k*_ = *n*(*n* − 1)⋯(*n* − *k* + 1) *is the falling factorial*.

*Proof*. There are *nf*(*n*) one-colorings in the full *n*-network, plus one origin. Each one of the one-colorings can be thought of as the origin of an *n* − 1-network, which in turn generate (*n* − 1)*f*(*n* − 1) one-colorings in an embedded *n* − 1-network, for a total of

$$\begin{array}{c} nf\left(n\right)\left(n-1\right)f\left(n-1\right) \end{array}$$

configurations using 1, 2, and perhaps 0, hence termed two-colorings. However, one of the *f*(*n*) one-colorings is the 0 state of the *n*-network, so it does not generate any two-colorings. Thus, there are at most 1 + *nf*(*n*) + *n*(*n* − 1)(*f*(*n*) − 1)*f*(*n* − 1) zero-, one-, and two-colorings. Now assume that the number of *k*-colorings is

$$\begin{array}{c} n\left(n-1\right)\cdots \left(n-k+1\right)\left(f\left(n\right)-1\right)\left(f\left(n-1\right)-1\right)\cdots \left(f\left(n-k+2\right)-1\right)f\left(n-k+1\right). \end{array}$$

Of these,

$$\begin{array}{c} n\left(n-1\right)\cdots \left(n-k+1\right)\left(f\left(n\right)-1\right)\left(f\left(n-1\right)-1\right)\cdots \left(f\left(n-k+2\right)-1\right) \end{array}$$

are origins of an *n* − *k*-network, meaning they are actually *k* − 1-colorings; they cannot generate any *k* + 1-colorings. The remaining

$$\begin{array}{c} n\left(n-1\right)\cdots \left(n-k+1\right)\left(f\left(n\right)-1\right)\left(f\left(n-1\right)-1\right)\\ \cdots \left(f\left(n-k+2\right)-1\right)\left(f\left(n-k+1\right)-1\right) \end{array}$$

are genuine *k*-colorings which can generate *f*(*n* − *k*) one-colorings in the *n* − *k*-network, or equivalently, *k* + 1-colorings. Thus, the total number of zero-, one-, two-, …, *k* + 1-colorings is no more than

$$\begin{array}{c} n\left(n-1\right)\cdots \left(n-k+1\right)\left(f\left(n\right)-1\right)\left(f\left(n-1\right)-1\right)\\ \cdots \left(f\left(n-k+2\right)-1\right)\left(f\left(n-k+1\right)-1\right). \end{array}$$

This induction argument proves the statement.

### 8. Properties of the orbits in the ratchet and sequestration network

First we define what it means to be an *origin* and an *orbit* in the threshold-1 ratchet network and determine the number of orbits as a function of *n* and *m*. Then we prove that the configurations in the sequestration network are defined by reversible paths.

A forbidden configuration in the ratchet network contains some row or column permutation of the pattern $\left(\begin{array}{cc} 1 & 0\\ 0 & 1 \end{array}\right)$ on any 2 × 2 sub-block of the connectivity matrix **A**. This is the minimum violation, but larger blocks may violate this pattern as well, for example $\left(\begin{array}{cc} 1 & 0\\ 0 & 1\\ 1 & 0 \end{array}\right)$ has 2 violations. Furthermore, application of any of the *K*’s or *P*’s in this sub-block will relieve at least one of these violations. Therefore, we define an *i*, *j*-*orbit* in the ratchet network as the locus of configurations having a forbidden configuration on an *i* × *j* sub-block that does not use the corresponding set of *i* *K*’s and *j* *P*’s. The *origin* of any *i*, *j*-orbit is the configuration having all remaining *nm* − *ij* entries of **A** equal to 0 (or all 1 to make the case of having only *P* actions symmetric with having only *K*’s). A matrix **X** having the same forbidden *i* × *j* sub-block as an origin **Y** is not considered to be in the orbit of **Y** if (i) there is no sequence of actions that transforms **Y** to **X**, or (ii) if the sequence involves one of the forbidden *K*’s or *P*’s. With these restrictions, the number of origins is equal to the number of orbits.

Denote by $c_{i}^{j}$ the number of orbits in a ratchet network of size *n* × *m* with violations involving *i* ≤ *n* *K*’s and *j* ≤ *m* *P*’s. If *i* = *j* = 2 there are $2^{ij}-B_{2}^{-2}=2$ forbidden configurations that turn into origins for the remaining *n* − *i* *K*’s and *n* − *j* *P*’s. There are more orbits in these smaller networks. For every *i*′, *j*′ ≥ 2 there are (ii′)(jj′)ci′j′Bi-i′-(j-j′) configurations reached by orbits using *i*′ *K*’s and *j*′ *P*’s. Only configurations *not* reached by these orbits are available as new origins when the number of *K*’s and *P*’s not to be used is *i* and *j*, respectively. Finally, there are (ni)(mj) ways to specify *i* ≤ *n* *K*’s and *j* ≤ *m* *P*’s. Then we have the

**Theorem 11** *For a given set of* *i* ≤ *n* *K’s and* *j* ≤ *m* *P’s, the number of* *i*, *j*-*orbits is*

$$c_{i}^{j}=\left(2^{ij}-B_{i}^{-j}\right)-\sum_{\begin{array}{c} i^{\prime },j^{\prime }\geq 2\\ i^{\prime }+j^{\prime }\leq i+j-1 \end{array}}^{i,j}\left(\begin{array}{c} i\\ i^{\prime } \end{array}\right)\left(\begin{array}{c} j\\ j^{\prime } \end{array}\right)c_{i^{\prime }}^{j^{\prime }}{B^{\prime }}_{i-i^{\prime }}^{-\left(j-j^{\prime }\right)},$$ (26)

*and the the total number of* *i*, *j*-*orbits in the* *n* × *m* *ratchet network is*

Cijn,m=nimjcij, (27)

*where*

$$\begin{array}{c} {B^{\prime }}_{i-i^{\prime }}^{-\left(j-j^{\prime }\right)}=\left\{\begin{array}{cc} B_{i-i^{\prime }}^{-\left(j-j^{\prime }\right)} & if\;i-i^{\prime }>0\;\text{and}\;j-j^{\prime }>0\\ 2^{i-i^{\prime }} & if\;j-j^{\prime }=0\\ 2^{j-j^{\prime }} & if\;i-i^{\prime }=0 \end{array}\right.. \end{array}$$ (28)

The modification *B*′ ensures that an orbit lacking allowable *P*’s (*K*’s) can still use *K*’s (*P*’s). A table of values of Eq (27) is given in S4 Fig.

We noted in the main text that configuration in the sequestration network can be joined by reversible paths. A path *K*_*i*_ *P*_*j*_ or *P*_*j*_ *K*_*i*_ is *reversible* if a configuration reached by the sequence of actions *w* is also reached by the either the sequence *wK*_*i*_ *P*_*j*_ or *wP*_*j*_ *K*_*i*_, but not *wK*_*i*_ or *wP*_*j*_, respectively. Thus we can also prove the

**Theorem 12** *There always exists a reversible path between any two configurations in an orbit of the sequestration network*.

*Proof*. Let **x** be a configuration in an orbit using *m* ≤ *n* of the actions, and let *P* denote the locus of configurations reached from **x**. We now need to show that *P* must be reversibly reached from the origin. Denote by $\bar{P}$ the complement of *P*, so that any $y\in \bar{P}$ is reversibly reached from the origin. In order for there to be no reversible path between **x** ∈ *P* and $y\in \bar{P}$, there must always be a state *i* such that *K*_*i*_ increases the number of targets {⋅, *i*} in the *i* state and *P*_*i*_ increases the number of targets {⋅, *i*} in the zero state. Now assume there is a configuration **z** ∈ *P* using all *m* allowed states. **z** must have at least one target in the 0 state, but this is un-allowed, because then **z** would violate the connection rule. Therefore, there is a maximum number *m*′ < *m* of states used by any **x** ∈ *P*. Now assume there is a configuration **z**′ ∈ *P* using all *m*′ allowed states. But this implies that there is a single-arm target {0, *j*} that must be in the zero state. Then the action *K*_*j*_ takes **z**′ to a configuration $y\in \bar{P}$ and *P*_*j*_ takes **y** to **z**. This path must be reversible, and **z**′ is reached reversibly from the origin. By induction we conclude that *m*′ = 0 and that *P* = Ø. Finally, because any two configurations are reached reversibly from the origin, there is a reversible path between them.

Theorem 12 defines the orbits of the sequestration network as those configurations connected by reversible paths.

### 9. A universal formulation of the actions as matrix operators

In this section we show how to write the *K* and *P* regulators as matrix operators in a manner consistent with both models considered in the paper. First we define the vector space V of configurations of the *N* targets, then we derive the operators that transform V.

Let x∈V. For a network with *N* targets we require that ∑_*i*_ *x*_*i*_ = *N*. This means that **x** has at least *N* entries, and in general dim **x** ≥ *N*. Therefore, we cannot use the standard state space of *N*-dimensional vectors, because the operators will not conserve the number of targets. Each target has a 0 state. The number *D* of independent directions accessible from 0 is called the *dimension* of the network, and the number *T* of steps one can move along each dimension is called the *threshold*. In the ratchet model, each target has a single ladder of states with variable threshold, so *D* = 1 and *T* is allowed to vary; in the sequestration model *D* = *n* but the threshold is *T* = 1.

Denote by *A*_*di*_ the fraction of the targets of type *A* in state *i* ∈ {0, 1, …, *T*} along dimension *d*. For a subset of the targets a *K*-type action causes population transfer between states (*d*, *j*) and (*d*, *i*) with *i* = *j* + 1, and a *P*-type action the reverse. If a *K* regulator acts for a short time we can write the “reaction rate” equation as

$$\begin{array}{cc} {\dot{x}}_{A_{j}} & =-g_{A}x_{A_{j}}\\ {\dot{x}}_{A_{i}} & =+g_{A}x_{A_{j}} \end{array}$$ (29)

where *g*_*A*_ > 0 is a proportionality constant. This defines a matrix differential equation

$$\begin{array}{c} \dot{x}=G_{dj}\cdot x \end{array}$$ (30)

with $x\in R^{N(DT+1)\times 1}$ the vector of populations of the *DT* + 1 states of the *N* targets and $G_{dj}\in R^{N(DT+1)\times N(DT+1)}$ the block diagonal matrix of rate constants between the *j* and *j* + 1 population states along dimension *d*. Eq (29) can be rewritten

$$\begin{array}{c} \left(\begin{array}{c} {\dot{x}}_{A_{j}}\\ {\dot{x}}_{A_{i}} \end{array}\right)=\left(\begin{array}{cc} -g_{A} & 0\\ g_{A} & 0 \end{array}\right)\cdot \left(\begin{array}{c} x_{A_{j}}\\ x_{A_{i}} \end{array}\right). \end{array}$$ (31)

Because **G**_**dj**_ is block diagonal, Eq (30) can be solved by exponentiation on each block:

$$\begin{array}{c} \left(\begin{array}{c} x_{A_{j}}\left(t\right)\\ x_{A_{i}}\left(t\right) \end{array}\right)=\exp\left\{\left(\begin{array}{cc} -g_{A} & 0\\ g_{A} & 0 \end{array}\right)t\right\}\cdot \left(\begin{array}{c} x_{A_{j}}\left(0\right)\\ x_{A_{i}}\left(0\right) \end{array}\right)=\left(\begin{array}{cc} e^{-g_{A}t} & 0\\ 1-e^{-g_{A}t} & 1 \end{array}\right)\cdot \left(\begin{array}{c} x_{A_{j}}\left(0\right)\\ x_{A_{i}}\left(0\right) \end{array}\right). \end{array}$$ (32)

The restriction of the model from a continuous range of population states *x*_*A*_*i*__ ∈ [0, 1] to the boolean values {0, 1} formally emerges by considering the “reaction” *K* catalyzes on its targets to have gone to completion. We do this by taking the the limit *t* → ∞ in Eq (32) to get

$$\begin{array}{c} \left(\begin{array}{c} x_{A_{j}}\left(t\right)\\ x_{A_{i}}\left(t\right) \end{array}\right)=\left(\begin{array}{cc} 0 & 0\\ 1 & 1 \end{array}\right)\cdot \left(\begin{array}{c} x_{A_{j}}\left(0\right)\\ x_{A_{i}}\left(0\right) \end{array}\right), \end{array}$$ (33)

so that the matrix **K**_**dj**_ defined by

$$\begin{array}{c} K_{dj}=\lim_{t\to \infty }\exp\left\{G_{dj}t\right\} \end{array}$$ (34)

is the block diagonal matrix having 1’s at (row, column) positions (1 + (*d* − 1)*T* + *i*, 1 + (*d* − 1)*T* + *j*) of each block that responds to *K* in dimension *d* and admits population transfer between from *j* to *i*.

Because *K* acts on all targets at once, it is insensitive to the initial state *j*. Thus the matrix corresponding to the action of *K* is

$$\begin{array}{c} K_{d}=\prod_{j}K_{dj}, \end{array}$$ (35)

which is the block diagonal matrix having 1’s at (row, column) positions

$$\begin{array}{c} \left(1+\left(d-1\right)T+1,1+\left(d-1\right)T+0\right),\ldots ,\left(1+\left(d-1\right)T+T,1+\left(d-1\right)T+T-1\right)\\ \text{and}\;\left(1+\left(d-1\right)T+T,1+\left(d-1\right)T+T\right) \end{array}$$

of each block that responds to *K* in dimension *d*.

This derivation can be repeated in the case that population goes in the opposite direction from at state *j* to a state *i* < *j* using a different set of rate matrices **H**_**dj**_ corresponding to the reverse of Eq (31). We obtain the block diagonal matrix **P**_**d**_ corresponding to the action of *P* in dimension *d* having 1’s at (row, column) positions

$$\begin{array}{c} \left(1+\left(d-1\right)T+0,1+\left(d-1\right)T+1\right),\ldots ,\left(1+\left(d-1\right)T+T-1,1+\left(d-1\right)T+T\right)\\ \text{and}\;\left(1+\left(d-1\right)T+0,1+\left(d-1\right)T+0\right) \end{array}$$

of each block that responds to *P* in dimension *d*. Whereas **K**_**d**_ is sub-diagonal, **P**_**d**_ is super-diagonal.

The Baker-Campbell-Hausdorf expansion shows that **K**_**d**_ in Eq (35) and in general any product of matrices **K**_**d**_ and **P**_**d**_ are generated by matrix exponentiation of commutators of the generators **G**_**dj**_, **H**_**dj**_. This is the origin of noncommutativity in both the ratchet and sequestration models.

An example in the sequestration network illustrates population transfer between states. In the *n* = 2 network on the targets *A*, *B*, and *C* the initial configuration of the network is represented by ${\left(\begin{array}{ccccccccc} A_{0} & A_{11} & A_{21} & B_{0} & B_{11} & B_{21} & C_{0} & C_{11} & C_{21} \end{array}\right)}^{T}={\left(\begin{array}{ccccccccc} 1 & 0 & 0 & 1 & 0 & 0 & 1 & 0 & 0 \end{array}\right)}^{T}$. Only targets *A* and *C* can access dimension 1, and only targets *B* and *C* can access dimension 2. Therefore the *t* → ∞ action of *K*_1_ on the network is given by

$$\begin{array}{c} \left(\begin{array}{ccccccccc} e^{-g_{A}t} & 0 & 0 & 0 & 0 & 0 & 0 & 0 & 0\\ 1-e^{-g_{A}t} & 1 & 0 & 0 & 0 & 0 & 0 & 0 & 0\\ 0 & 0 & 0 & 0 & 0 & 0 & 0 & 0 & 0\\ 0 & 0 & 0 & 1 & 0 & 0 & 0 & 0 & 0\\ 0 & 0 & 0 & 0 & 0 & 0 & 0 & 0 & 0\\ 0 & 0 & 0 & 0 & 0 & 1 & 0 & 0 & 0\\ 0 & 0 & 0 & 0 & 0 & 0 & e^{-g_{C}t} & 0 & 0\\ 0 & 0 & 0 & 0 & 0 & 0 & 1-e^{-g_{C}t} & 1 & 0\\ 0 & 0 & 0 & 0 & 0 & 0 & 0 & 0 & 1 \end{array}\right)\left(\begin{array}{c} 1\\ 0\\ 0\\ 1\\ 0\\ 0\\ 1\\ 0\\ 0 \end{array}\right)\underset{t\to \infty }{\to }\left(\begin{array}{c} 0\\ 1\\ 0\\ 1\\ 0\\ 0\\ 0\\ 1\\ 0 \end{array}\right). \end{array}$$ (36)

Only *A* and *C* advance to state 1 and the number of targets (3) is conserved.

## Supporting Information

S1 Fig Scaling in combinatorial networks is sub-exponential. **(A)** An example network with *u* = 3 pools of *n* = 2 regulators each. A target is only ON if all *u* of its regulators bind. **(B)** Plots of Eq (18) vs. *n* for an increasing number of pools *u* and increasing redundancy *l*_*n*_.
(TIF)

S2 Fig Number of unique words in the threshold 1 ratchet network as a function of *n*, *m*, *l*_*n*_, *and l*_*m*_ found using Eq (24). *n* and *m* increase the across the rows and up the columns. *l*_*n*_ and *l*_*m*_ increase down the columns and across the rows of the sub-blocks.
(TIF)

S3 FigThe full *n*-network model has upper and lower bounds.**(A)** A plot of all the allowed configurations of a set of targets controlled by *n* = 3 regulators pairs in the full *n*-network. Blue, cyan, yellow, and red correspond to states 0, 1, 2, and 3, respectively. **(B)** A list of the words generating the corresponding states in **A**. *K* actions are shown in the red spectrum, and *P* in the blue. **(C)** A logarithmic plot of the bounds on the full model. The total space is ∏i=0n(i+1)(ni), the upper and lower bounds are calculated from Eqs (24) and (7), respectively, and the combinatorial model is 2^2*n*^.(TIF)

S4 Fig Number of orbits restricted from using *i* of the *K*’s and *j* of the *P*’s in the threshold 1 ratchet network as a function of *n* and *m* calculated using Eq (27). *n* and *m* increase the across the rows and up the columns. *i* and *j* increase down the columns and across the rows of the sub-blocks.
(TIF)
