# Supplementary figures and images for: Noncommutative Biology: Sequential Regulation of Complex Networks
Source: PLoS Comput Biol. 2016 Aug 25;12(8):e1005089. doi: 10.1371/journal.pcbi.1005089 (PMC4999240; doi:10.1371/journal.pcbi.1005089)

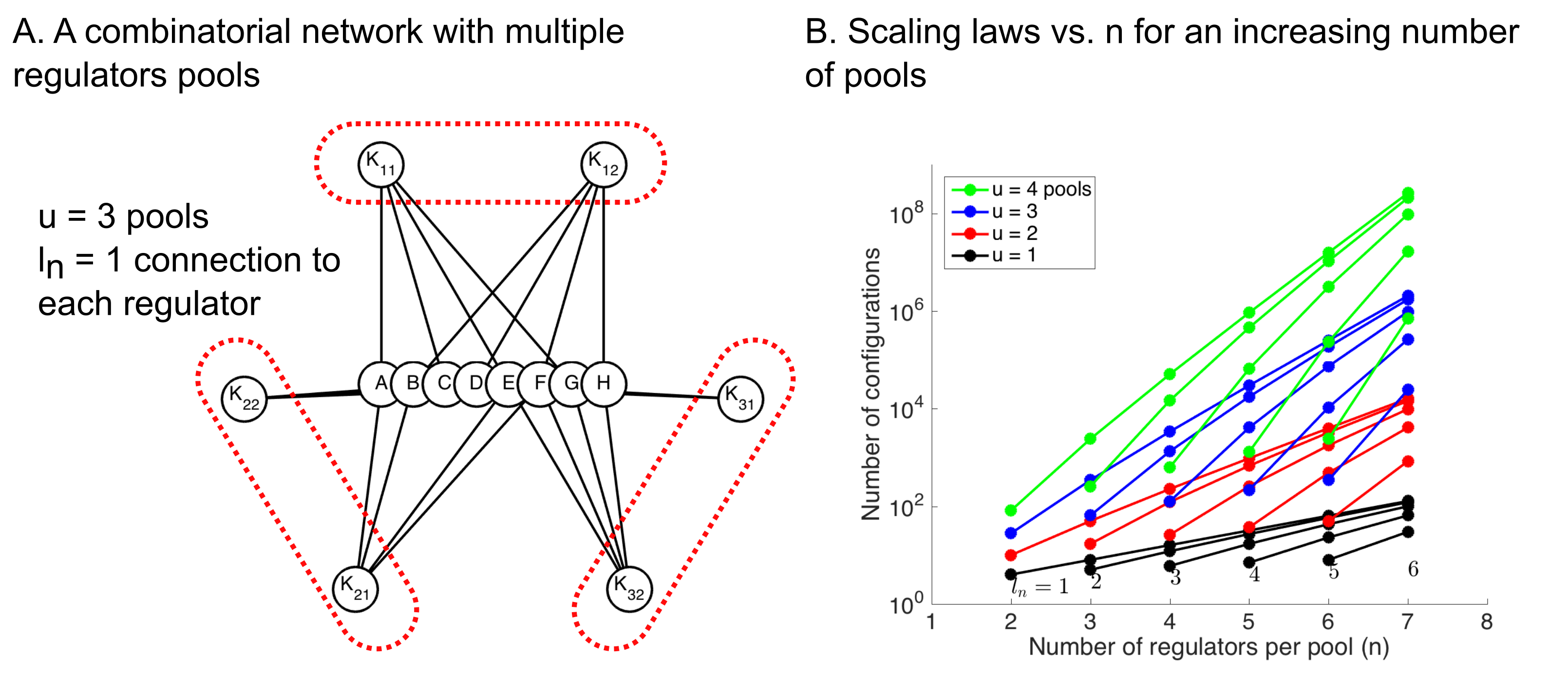

Supplement: S1 Fig — (A) An example network with u = 3 pools of n = 2 regulators each. A target is only ON if all u of its regulators bind. (B) Plots of Eq (18) vs. n for an increasing number of pools u and increasing redundancy ln. (TIF) [file pcbi.1005089.s001.tif]

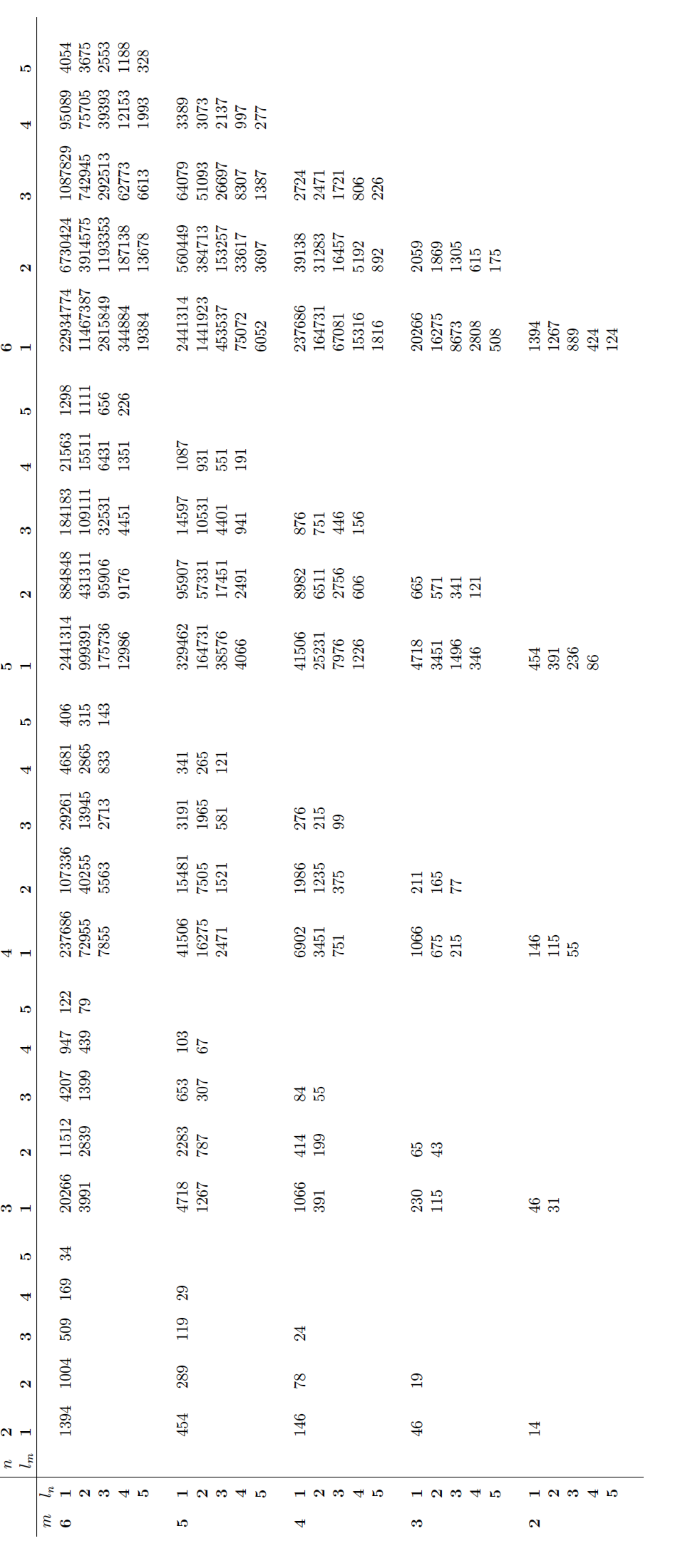

Supplement: S2 Fig — n and m increase the across the rows and up the columns. ln and lm increase down the columns and across the rows of the sub-blocks. (TIF) [file pcbi.1005089.s002.tif]

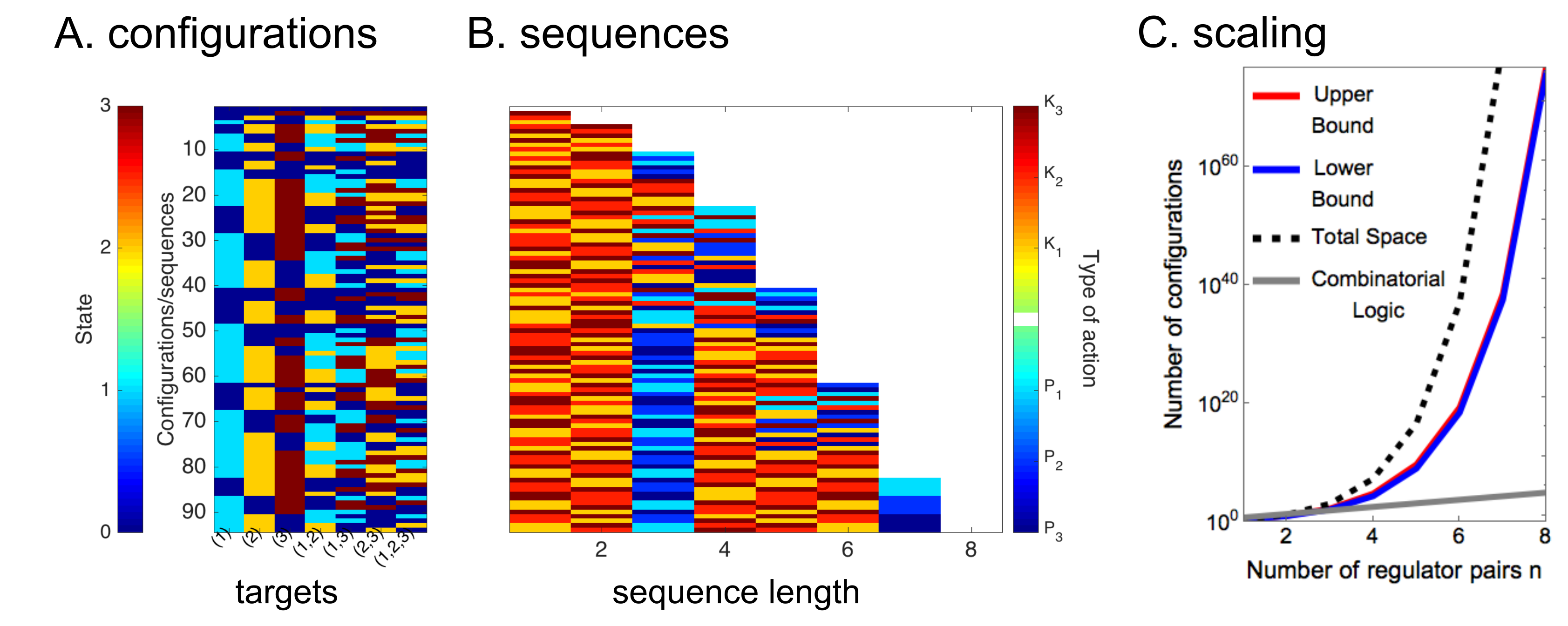

Supplement: S3 Fig — (A) A plot of all the allowed configurations of a set of targets controlled by n = 3 regulators pairs in the full n-network. Blue, cyan, yellow, and red correspond to states 0, 1, 2, and 3, respectively. (B) A list of the words generating the corresponding states in A. K actions are shown in the red spectrum, and P in the blue. (C) A logarithmic plot of the bounds on the full model. The total space is ∏i=0n(i+1)(ni), the upper and lower bounds are calculated from Eqs (24) and (7), respectively, and the combinatorial model is 22n. (TIF) [file pcbi.1005089.s003.tif]

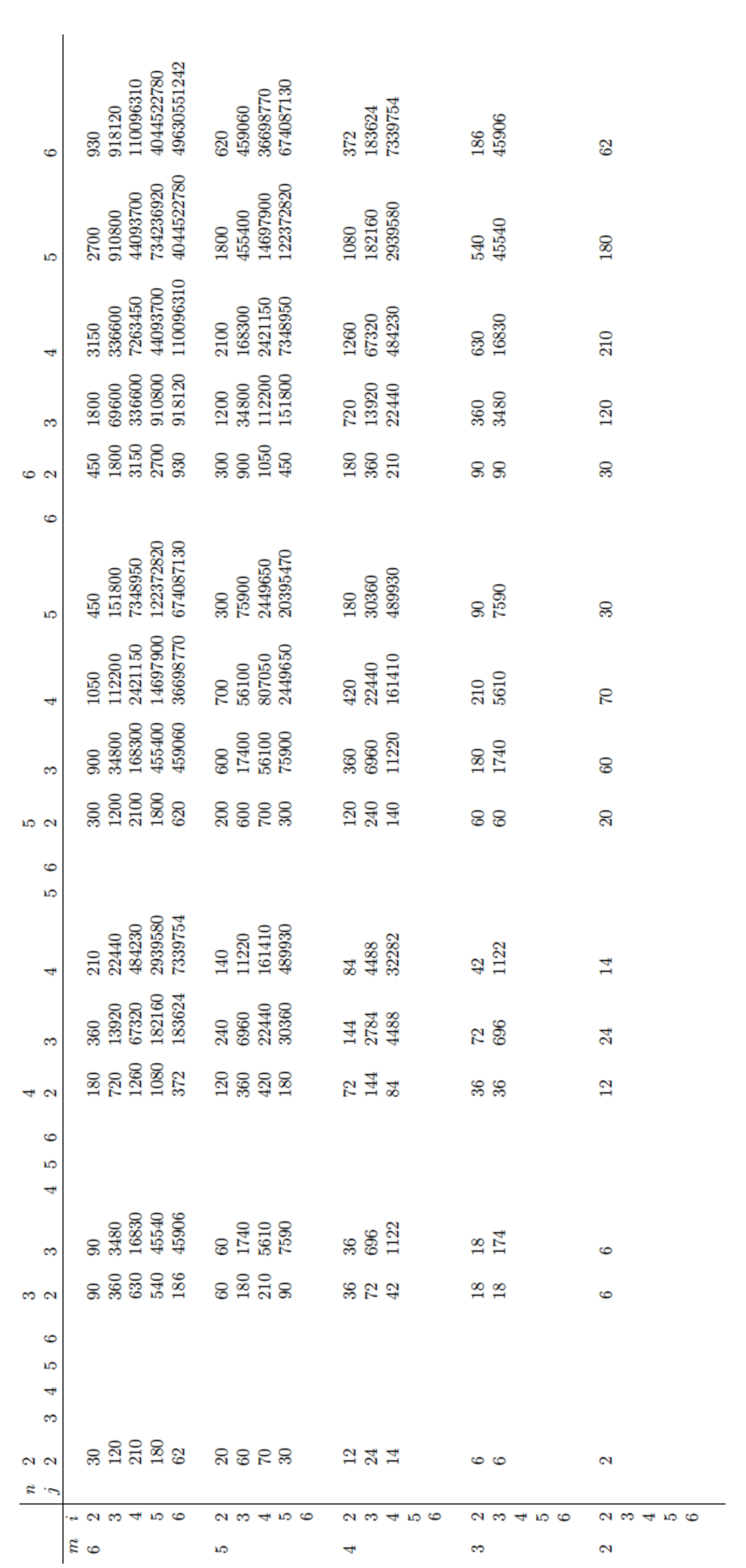

Supplement: S4 Fig — n and m increase the across the rows and up the columns. i and j increase down the columns and across the rows of the sub-blocks. (TIF) [file pcbi.1005089.s004.tif]
